# Regulatory, clinical, and post-marketing challenges of lecanemab for Alzheimer’s disease: insights from real-world data

**DOI:** 10.1007/s10072-026-08829-4

**Published:** 2026-03-06

**Authors:** Giuseppe Marano, Roberto Da Cas, Ilaria Ippoliti, Paolo Caffarra, Nicoletta Locuratolo, Nicola Vanacore, Antonio Ancidoni

**Affiliations:** 1 National Centre for Drug Research and Evaluation, Italian National Institute of Health, Via Giano Della Bella 34, 00162, Rome, Italy; 2 Member of the National Committee on Dementia of the National Dementia Plan, Rome, Italy; 3 National Centre for Disease Prevention and Health Promotion, Italian National Institute of Health, Via Giano Della Bella 34, 00162 Rome, Italy

**Keywords:** Alzheimer’s disease, Mild Cognitive Impairment, Lecanemab, Real-world evidence, Safety, Pharmacovigilance, FAERS

## Abstract

**Introduction:**

In recent years, lecanemab received regulatory approval from several regulatory agencies. The safety profile, particularly the risk of amyloid-related imaging abnormalities (ARIA), necessitates post-marketing surveillance. From a public health perspective, generating robust real-world evidence (RWE) is essential.

**Objectives:**

This study aims to inform policy and clinical decision-makers by analyzing prescribing information, literature evidence, and the FDA Adverse Events Reporting System (FAERS) pharmacovigilance reports.

**Methods:**

This study employed a mixed-method approach. First, prescribing information for lecanemab was collected and compared across four regulatory agencies. Second, a systematic literature review was conducted in MEDLINE and Embase to identify RWE studies reporting adverse events (AEs), symptoms, or management strategies in patients treated with lecanemab. Finally, post-marketing safety data from the FAERS database were analyzed.

**Results:**

Four regulatory agencies have approved lecanemab through different pathways, each requiring confirmation of amyloid pathology and careful assessment of ARIA risk, particularly in Apolipoprotein E (ApoE) ε4 homozygotes. Notable differences exist across agencies regarding indications, contraindications, monitoring protocols, and criteria for treatment suspension, resumption, or discontinuation. All authorities mandate post-marketing programs to ensure ongoing monitoring of safety and effectiveness.

A bibliographic search identified 26 studies. Nine cohort studies included between 19 and 407 participants and reported follow-up periods ranging from 6 to 14 months; in a few studies, lecanemab was administered to individuals with moderate or severe AD. As expected, infusion-related reactions (IRRs) and ARIA were the most frequent adverse events, predominantly occurring within the first seven infusions. Some studies reported preliminary efficacy outcomes, although attrition bias may have affected these findings. Seventeen case reports described nineteen individuals aged 57–82, with most AEs arising between the 3rd and 7th infusion and primarily consisting of ARIA; serious events such as stroke, seizures, and two fatalities were also noted. In most cases, lecanemab was paused or permanently discontinued.

Analysis of the FAERS database identified 1,286 reports revealing 2,627 AEs, of which 30% were classified as serious, including forty-six deaths. The most reported AEs were headache, ARIA-E, ARIA-H, and chills. ARIA-E and ARIA-H have similar demographics, onset timing, and severity profiles.

**Conclusion:**

This study highlights the complexity of lecanemab’s safety profile and the variability in regulatory prescribing recommendations. While ARIA, especially in ApoE ε4 homozygotes, remains the most frequent adverse event, its severity ranges from mild to, in rare cases, severe or fatal. These findings underscore the need for robust post-marketing surveillance and harmonized recommendations to ensure safe and effective clinical use.

**Supplementary Information:**

The online version contains supplementary material available at 10.1007/s10072-026-08829-4.

## Introduction

In the class of new disease-targeting therapies (DTT) for Alzheimer's disease (AD), amyloid-targeting therapies (ATT) such as monoclonal antibodies (mAbs) are currently being investigated for their potential contribution to dementia care and prevention strategies [1]. In recent years, regulatory authorities granted marketing authorisation for amyloid-targeting mAbs to treat mild cognitive impairment (MCI) or mild dementia due to AD. However, the risk–benefit profile of these drugs remains highly controversial. In 2021, the Food and Drug Administration (FDA) granted conditional approval for aducanumab [2], while the European Medicines Agency (EMA) rejected the application due to insufficient clinical evidence of benefit [3]. In 2023, lecanemab was approved by the FDA via an accelerated regulatory pathway and subsequently received full approval [4]. After re-examining its initial refusal, the EMA recommended marketing authorisation for lecanemab in November 2024 [5] and for donanemab in July 2025 [6]. In the UK, lecanemab and donanemab have been authorised by the Medicines and Healthcare products Regulatory Agency (MHRA) [7, 8]. However, following cost-effectiveness assessments, the National Institute for Health and Care Excellence (NICE) issued two guidance documents indicating that the two drugs are not recommended for use within the National Health Service (NHS) in England [9, 10]. This highlights a discrepancy between regulatory approval by the MHRA and NICE’s cost-effectiveness evaluations.

Despite differing decisions and varying complexities in the approval procedures, mAbs have reached or are close to reaching the market. However, their safety profile, as observed in clinical trials, warrants close monitoring. ATT can cause amyloid-related imaging abnormalities (ARIA), including ARIA with edema (ARIA-E) and ARIA with haemosiderosis/microhaemorrhages (ARIA-H) [11–15].

In the CLARITY-AD trial on lecanemab, the incidence of ARIA-E was 12.6% and ARIA-H occurred in 17% of patients. Notably, higher risks of ARIA were observed in Apolipoprotein E (ApoE) ε4 carriers, especially homozygotes [13]. These findings highlight variability in ARIA incidence and emphasize the need for close monitoring, particularly in higher-risk populations.

Therefore, collecting real-world evidence (RWE) is essential to assess the effectiveness and safety of these mAbs thoroughly. Initial efforts to describe post-marketing data using the FDA Adverse Event Reporting System (FAERS) database have recently been published, especially on lecanemab [16–22]. Data revealed notable adverse events (AEs), including ARIA-E and infusion-related reactions (IRRs). Serious adverse events (SAEs) and fatalities were also reported.

Therefore, this study aims to collect data on lecanemab from RWE through three different perspectives:

1. The regulatory perspective, analysing lecanemab prescribing information from four regulatory authorities;
2. The clinical perspective, reviewing literature from RWE studies to examine the clinical management of patients receiving lecanemab;
3. The pharmacovigilance perspective, investigating AEs associated with lecanemab in the post-marketing phase using the FAERS database [23].

## Materials and methods

### Analyses of regulatory pathways

The first analysis aimed to collect and compare the prescribing information for lecanemab by four regulatory authorities. The analysis focused on documents issued by the FDA for the United States, the EMA for European Union countries, the MHRA for the United Kingdom, and the Pharmaceuticals and Medical Devices Agency (PMDA) for Japan. The four agencies have been included considering their relevance in the international context and the availability of a public summary of product characteristics (SmPC). Prescribing information was retrieved from the official websites of these regulatory bodies or other publicly accessible regulatory databases [24–27]. Key elements of each document were evaluated, including approved indications, dosing regimens, safety profiles, contraindications, and monitoring requirements. The comparative analysis sought to identify patterns and variations in regulatory decisions and clinical guidance.

### Literature review

This systematic literature review aimed to identify and analyse published, peer-reviewed studies on the clinical and safety outcomes associated with lecanemab in the real-world clinical practice. A comprehensive literature search was conducted in two major biomedical databases, MEDLINE and Embase, for studies published in English between 2023, the year of the FDA approval of lecanemab, and November 16th, 2025. To maximize sensitivity and inclusiveness, a broad search strategy was intentionally employed using the terms “lecanemab” OR “leqembi”. Eligible studies included case reports and real-world cohort studies investigating clinical and safety outcomes of lecanemab, providing data on clinical manifestations and therapeutic or management approaches.

Exclusion criteria were: i) articles published in languages other than English; ii) studies based on administrative healthcare databases; iii) surveys that collected perceptions on lecanemab from patients, caregivers, or healthcare professionals; iv) studies exclusively evaluating other mAbs approved for AD (e.g., aducanumab or donanemab); v) studies on lecanemab reporting solely biomarker-based outcomes. Conference abstracts, even when relevant to the topic, and preprint articles not yet accepted for publication were also excluded.

Data were extracted and summarized in separate tables according to study design. For cohort studies, disaggregated data on AEs were reported whenever available, including information on individuals who experienced AEs and management approaches. For case reports, each clinical presentation was described in detail within a dedicated table.

Tables included the following variables: country of data collection, number of included participants (for cohort studies), clinical and demographic characteristics, including disease stage, genotype, history or incidental comorbidities. Furthermore, a detailed description of AEs and related symptoms, follow-up outcome(s), and status of drug administration were also collected (Tables S1 and S2). The quality of included studies was assessed by using appraisal tools from the Johanna Briggs Institute (https://jbi.global/critical-appraisal-tools). For cohort studies, the checklist for prevalence/incidence studies was applied to evaluate the reporting of ARIA events as the main outcome. All items of the checklists and the evaluations are available in Tables S7 and S8.

### The FAERS database

Established to support the FDA’s post-market safety surveillance of pharmaceuticals and biologics, the FAERS database systematically aggregates suspected AEs and medication errors reported by healthcare professionals, patients, and pharmaceutical manufacturers, globally [23, 28]. The FAERS dataset comprises demographic information (DEMO), drug details (DRUG) and adverse events (REAC). These datasets can be linked through a primary key, the “PRIMARYID”. All reports where lecanemab was indicated as "primary suspect drug" were extracted from the FAERS database considering the period from January 1, 2021 to December 31 st, 2024 (n = 1,565). One hundred and five duplicated reports were excluded. Reports submitted by countries different from the USA and Japan (n = 42), reports containing AEs that occurred before lecanemab administration (n = 115) or before the first FDA approval on January 6, 2023 (n = 12) or reports from literature (n = 5) were excluded. The Medical Dictionary for Regulatory Activities (MedDRA) was used to standardize the classification of AEs into Preferred Terms (PTs) [29]. An analysis of the distribution characteristics of lecanemab AE reports, focusing on SAEs and ARIA events, was carried out. To evaluate the temporal patterns of AEs associated with lecanemab, a time-to-onset analysis was performed. The time-to-onset is a useful metric but has some limitations in spontaneous reports, such as an inaccurate definition of dates (administration or starting therapy, onset of AEs), notifying delays or reporter’s attitude. The lack of information in the FAERS database doesn’t allow for robust inferential analysis. Ethical approval was not required for this study because it used the FAERS database, which is a free, open-access database.

## Results

### Prescribing information from regulatory authorities

Table 1 summarizes the four regulatory assessments. Lecanemab followed different approval pathways: the FDA granted accelerated and then full approval based on CLARITY-AD data [13]; the PMDA completed a priority review in eight months; EMA and MHRA required full Phase 3 data before approval.

**Table 1:** Prescribing information descriptions from the included regulatory authorities

	FDA Prescribing information			PMDA Deliberation of results			MHRA Prescribing information			EMA Prescribing information		
Country or Region	United States			Japan			United Kingdom			European Union		
Regulatory approval pathway	Accelerated approval followed by traditional approval			Priority review			Traditional approval			Traditional approval		
Year of approval	July 2023 (prescribing information revised in January 2025)			August 2023			August 2024 (revised in February 2025)			April 2025		
Therapeutic indications*	For AD. Treatment should be initiated in patients with MCI or mild dementia stage of disease			To slow the progression of MCI and mild dementia due to AD			Indicated for the treatment of MCI and mild dementia due to AD			Clinical diagnosis of MCI or mild dementia due to AD		
Patient selection	• Patients with confirmed amyloid pathology (not clearly defined the assessment method) • ApoEε4 homozygotes **can receive** lecanemab following discussion regarding ARIA risks across ApoE genotypes • ApoEε4 heterozygotes and noncarriers **can receive** lecanemab			• Patients with confirmed amyloid pathology as assessed by PET or CSF • ApoEε4 homozygotes **can receive** lecanemab if strict risk management for ARIA is implemented • ApoEε4 heterozygotes and noncarriers **can receive** lecanemab			• Patients with confirmed amyloid pathology as assessed by PET or CSF • Lecanemab is **not recommended** in ApoEε4 homozygotes • ApoEε4 heterozygotes and noncarriers **can receive** lecanemab			• Patients with confirmed amyloid pathology (not clearly defined the assessment method) (not specified the assessment method) • Lecanemab is **not recommended** in ApoEε4 homozygotes • ApoEε4 heterozygotes and noncarriers **can receive** lecanemab		
Contraindications	• People with serious hypersensitivity to active treatment or excipients • **Consider caution** in people on anticoagulant therapy due to increased risk of intracerebral haemorrhage			• Presence of vasogenic cerebral edema • ≥ 5 cerebral MHs, superficial siderosis, or > 1 cm cerebral haemorrhage • Antithrombotic drugs (i.e., anticoagulants) should be listed in the “*precautions for co-administration*”			• Serious hypersensitivity to active treatment or excipients • Pre-treatment MRI findings of prior intracerebral haemorrhage, > 4 MHs, superficial siderosis or vasogenic edema, which are suggestive of CAA • Ongoing anticoagulant therapy			• Hypersensitivity to the active substance or excipients • Bleeding disorders that are not under adequate control • Pre-treatment MRI findings of prior intracerebral haemorrhage, > 4 MHs, superficial siderosis or vasogenic oedema, or other findings, which are suggestive of CAA • Ongoing anticoagulant therapy		
Dosage and administration	Intravenous use • Biweekly (10 mg/kg) for 18 months • Monthly maintenance dose (10 mg/kg) after 18 months			Intravenous use • Biweekly (10 mg/kg)			Intravenous use • Biweekly (10 mg/kg)			Intravenous use • Biweekly (10 mg/kg)		
Discontinuation criteria	n/r			Treatment discontinuation if clinically ineffective (assessed every 6 months) or if disease progresses to a moderate/severe stage			Progression to moderate AD			• Progression to moderate AD; • If the clinical course suggests that lecanemab has not demonstrated effectiveness (cognitive symptoms should be assessed every 6 months)		
	• Recent MRI prior initiating lecanemab to identify preexisting ARIA • MRI prior to the 5th, 7th, and 14th infusions • If patient experiences symptoms suggestive of ARIA, clinical evaluation should be performed, including an MRI if indicated			n/r			• Recent MRI prior initiating lecanemab to identify preexisting ARIA • MRI prior to the 5th, 7th, and 14th infusions • Enhanced clinical vigilance for ARIA during the first 14 weeks • If the patient experiences symptoms suggestive of ARIA, clinical evaluation should be performed, including an MRI if indicated			• Recent MRI (6 months) prior initiating lecanemab to identify preexisting ARIA • Enhanced clinical vigilance for ARIA during the first 14 weeks • MRI prior to the 5th, 7th, and 14th infusions • Additional MRI may be needed at any time if patients present symptoms suggestive of ARIA		
		**ARIA-E**	**ARIA-H**		**ARIA-E**	**ARIA-H**		**ARIA-E**	**ARIA-H**		**ARIA-E**	**ARIA-H**
Monitoring for ARIA and indications for dosing interruption	MRI severity **mild** and **asymptomatic**	May continue dosing	May continue dosing	MRI severity **mild** and **asymptomatic**	n/r	n/r	MRI severity **mild** and **asymptomatic**	May continue dosing on clinical judgement	May continue dosing on clinical judgement	MRI severity **mild** and **asymptomatic**	May continue dosing on clinical judgement	May continue dosing on clinical judgement
	MRI severity and symptoms: **mild**	May continue dosing on clinical judgement	Suspend dosing	MRI severity and symptoms: **mild**	n/r	n/r	MRI severity and symptoms: **mild**	Suspend dosing	Suspend dosing	MRI severity and symptoms: **mild**	Suspend dosing	Suspend dosing
	MRI severity and symptoms: **moderate**	Suspend dosing	Suspend dosing	MRI severity and symptoms: **moderate**	n/r	n/r	MRI severity and symptoms: **moderate**	Suspend dosing	Suspend dosing	MRI severity and symptoms: **moderate**	Suspend dosing	Suspend dosing
	MRI severity and symptoms: **severe**	Suspend dosing	Suspend dosing	MRI severity and symptoms: **severe**	n/r	n/r	MRI severity and symptoms: **severe**	Suspend dosing	Permanently discontinue treatment	MRI severity and symptoms: **severe**	Suspend dosing	Permanently discontinue treatment
Post-marketing programmes	**Post-marketing pharmacovigilance with biannual reports to characterize:** • the risk of ARIA (E and H), along with any incident cerebral haemorrhage greater than 1 cm in size; • cases of vasculitis; • the risk of infusion reactions			**Additional pharmacovigilance activities** • Early post-marketing vigilance; • Specified use-results survey (long-term use); • Post-marketing clinical study;			• Activation of a controlled access programme			• Activation of an EU-wide *controlled access programme* to promote the safe and effective use of lecanemab and prevent off-label use; • Post-authorisation measures include the EU lecanemab All-Patient Study with annual reports beginning from September 2026; • Provision of a company’s guide and checklist for HCPs, an alert card for patients and training programmes on ARIA for HCPs; • Post-authorisation safety study to further characterize ARIA-E and ARIA-H		
				Additional risk minimization activities • Disseminate data gathered during early post-marketing phase vigilance; • Confirmation of proper use • Prepare and distribute; information materials for HCPs (proper use guide); • Prepare and distribute information materials for patients								

AD: Alzheimer’s disease; ApoE: Apoliprotein E; ARIA: Amyloid-related imaging abnormalities; CAA: cerebral amyloid angiopathy; CSF: cerebrospinal fluid; EMA: European Medicines Agency; EU: European Union; FDA: Food and Drug Administration; HCPs: Healthcare professionals; MCI: Mild cognitive impairment; MHRA: Medicines and Healthcare products Regulatory Agency; MHs: microhaemorrhages; MRI: Magnetic Resonance Imaging; PET: Positron emission tomography; PMDA: Pharmaceuticals and Medical Devices Agency; UK: United Kingdom; US: United States

^*^Therapeutic indications are those reported in the official reports of the regulatory authorities

n/r: not reported

Lecanemab is indicated for individuals with MCI or mild dementia due to AD, and confirmation of amyloid pathology is required before treatment begins. Although the FDA [24] and EMA [25] do not clearly recommend an assessment method, the MHRA [26] and PMDA [27] explicitly recommend using CSF analysis or amyloid PET. Testing for the ApoE genotype is required before starting treatment. While the FDA [24] and PMDA [27] recommend lecanemab in homozygotes under strict management schedules, the EMA [25] and MHRA [26] recommend against it in this group.

Contraindications differ across agencies: the FDA [24] lists only hypersensitivity, while the MHRA [26] and EMA [25] recommend against its use in patients with specific MRI findings or on anticoagulants. The PMDA [27] does not contraindicate antithrombotic therapy; instead, it recommends that such treatments be included under precautions for use.

The FDA [24] also recommends caution in patients with cerebral amyloid angiopathy (CAA)-related MRI findings or risk factors for intracerebral haemorrhages.

Lecanemab should be administered intravenously with biweekly infusions. Currently, only the FDA [24] has approved a monthly maintenance dose after 18 months of treatment. The effects of stopping treatment and the efficacy of lecanemab in later stages of AD remain unknown. According to the PMDA [27], efficacy should be regularly monitored, with an initial assessment at one year and subsequent evaluations every six months. Discontinuation should be considered if clinical data indicate a lack of efficacy or if the disease progresses to moderate or severe stages. Similarly, the EMA [25] recommends stopping treatment when the clinical course suggests that lecanemab has not shown effectiveness. The EMA [25] recommends cognitive assessments every six months. The MHRA [26] recommends discontinuing treatment in case of disease progression without providing clear guidance on how and when to evaluate it. Conversely, the FDA [24] does not specify any criteria for discontinuation.

The FDA, MHRA, and EMA [24–26] recommend specific schedules for monitoring ARIA. The three regulatory authorities recommend obtaining a recent MRI before initiating treatment to identify preexisting ARIA. MRI should be obtained before the 5th, 7th, and 14th infusions. Interestingly, MRI schedules do not differ between ApoEε4 carriers and non-carriers. Although the FDA [24] recommends the use of lecanemab, albeit with caution, in homozygotes, it does not advocate for a more intensive MRI schedule. Although the FDA, EMA and MHRA [24–26] recommend the use of additional MRI in case of symptoms suggestive of ARIA, the MHRA and EMA [25, 26] emphasise the importance of increased clinical vigilance during the first 14 weeks of treatment.

According to the FDA [24], lecanemab may be continued in patients who experience asymptomatic ARIA-E or ARIA-H and mild ARIA-E (upon clinical judgment). In case of ARIA-E, radiographically and clinically moderate/severe and ARIA-H radiographically and clinically mild, lecanemab should be suspended until MRI demonstrates radiographic resolution and symptoms resolve. The MHRA and EMA [25, 26] recommend that lecanemab should only be continued when cases of ARIA-E or ARIA-H are mild on radiographic imaging and no symptoms are present. If ARIA-H is severe on imaging and accompanied by severe symptoms, lecanemab should be permanently discontinued.

All four regulatory agencies mandate that the manufacturer implement specific post-marketing programs. The FDA [24] requires two biannual post-marketing reports to characterize AEs and recommends that physicians inform patients about the availability of a voluntary national registry and encourage their participation. The PMDA [27] requires conducting a post-marketing clinical study and early post-marketing vigilance. Moreover, to confirm safety and efficacy in clinical use, a specific use-result survey is required. The development of information materials for healthcare professionals and patients, along with the dissemination of data from the early post-marketing phase, are also mandated responsibilities for the manufacturer to mitigate risks. The MHRA and EMA [25, 26] recommend the activation of controlled access programs.

### Clinical and safety outcomes of lecanemab in RWE studies

Bibliographic search identified 1,897 records. After duplicate removal and screening, 48 articles were assessed for eligibility. Finally, 26 articles were included, nine cohort studies [30–38] and 17 case reports [39–55] (Tables S1, S2, S5 and Figure S1). Quality assessments of included studies are reported in Tables S7 and S8.

#### Cohort studies

Among nine included studies, four were conducted in the US [34–37], three in China [31–33], and one each in Israel and Japan [30, 38]. Five studies had a retrospective design [30, 34–37], while four were conducted prospectively [31–33, 38]. Three studies were multicentre [31, 33, 35], whereas six were single-centre [30, 32, 34, 36–38]. Settings were predominantly highly specialized, and most of the studies were conducted within academic institutions or in hospitals’ departments. Only a single study reported experience in a private neurological practice [35]. The sample size varied widely, ranging from 19 [34] to 407 participants [33]. Follow-up ranged between six and 14 months. Female sex ranged from 40% to 73.5%. Most studies included participants with a mean age between 72 and 74 years, consistent with the CLARITY-AD trial [13]. In contrast, three studies [31–33], all conducted in China, enrolled younger participants (mean age: 68.2 years). Most of the studies considered the Clinical Dementia Rating (CDR)-Global Score as a criterion to select participants. Two studies reported the clinical criteria according to the National Institute on Aging-Alzheimer’s Association (NIA-AA) to include participants, while two studies [34, 36] considered the experts’ appropriate use recommendations [57] to select potential treatment candidates. Confirmation of amyloid pathology was always mandatory in all studies; however, for those studies reporting the information, only one study [31] required amyloid PET, while the other studies considered both PET and CSF as confirmatory testing. Only one study did not report the clinical diagnosis of the included participants [34]. Regarding the other eight studies, the diagnosis of MCI was prevalent in four studies (range: 58.7%−89.1%) [32, 33, 36, 38], in line with the CLARITY-AD study. Conversely, in the other four studies [30, 31, 35, 37], most participants had a diagnosis of mild AD dementia (range: 39%–60%). Notably, two studies [31, 33] included 22.1% and 7.9% of participants with moderate AD dementia (as classified by the CDR-GS), and one study [31] reported that 1.5% of participants had severe dementia (CDR = 3). ApoEε4 carriers were prevalent in all studies (heterozygotes, range: 42%−62.5%; homozygotes, range: 4.7%−13%), while only one study [30] did not report genotype information. A single study excluded homozygotes as per protocol [38]. In the studies reporting information on the use of symptomatic treatment, lecanemab was used as add-on therapy since the majority of participants were receiving acetylcholinesterase inhibitors (range: 70.5%−97%) or memantine (range: 18%−66%). Further details and comparisons between the CLARITY-AD trial [13] and the included studies are reported in Table S5.

All included studies were primarily focused on examining the safety and feasibility of lecanemab in a clinical setting. However, only three studies considered cognitive and non-cognitive outcomes [31–33] while one study considered the rate of change in the CDR-SB as a preliminary analysis [36]. A single study [34] evaluated the effect of a genotype-guided slow titration and its effect on reducing the incidence of ARIA, while another study [30] assessed the safety of switching from lecanemab to donanemab in a cohort of 20 participants. A single study conducted in the US evaluated lecanemab administration across two clinical settings: an academic institution and a private neurology practice [35]. The authors compared these settings with respect to demographic characteristics, mean age, stage of clinical diagnosis, safety outcomes, and out-of-pocket costs associated with lecanemab infusions.

Dropout rates were reported in six studies [31–33, 35, 36, 38]. Dropouts (treatment-related or due to other causes) varied significantly, ranging from less than 10% in two studies [35, 36] to more than 80% after 9 months of treatment in a single study [33]. Dropouts, when reported, were mostly treatment-related, especially due to ARIA (see Table S5). Incident ARIA-E following treatment ranged from 3.1% to 15%, and isolated ARIA-H ranged from 4.4% to 10.9%. Three studies reported the incidence of ARIA-E + ARIA-H that ranged from 3.7% to 13.2%. IRRs ranged from 5.9% to 40%. In one study [37]**,** IRRs occurred in 37% of patients after the 1 st infusion, 7% after the 2nd infusion, 4–6% between the 3rd and 7th infusions, and none until the last injection (14th).

The risk of ARIA stratified by ApoE ε4 genotype was clearly reported only in three studies [32, 36, 37]. As expected, in all three US studies [35–37], the risk of ARIA-E increased with a higher number of ε4 alleles. However, one of these studies [36] reported that ARIA-H was not associated with the number of ε4 alleles, but with higher numbers of microhaemorrhages at baseline. Interestingly, in two studies from China, one reported no significant differences in the risk of ARIA among ApoE genotypes [33], while the other one [32] observed a higher incidence of ARIA-E in ApoE non-carriers than heterozygotes and no events in homozygotes.

Six out of nine studies reported data on clinical outcomes [30–33, 36, 38]. One study [30] observed that 17 out of 20 patients remained stable as measured by the CDR-GS. In a multicentre study conducted in China [31], after a follow-up of seven months, all clinical outcomes considered (i.e., ADAS-Cog14, CDR-SB, MoCA, MMSE) showed improvement; however, the analyses considered only 50% (n = 34) of the initial cohort. In the other two Chinese studies [32, 33], cognitive scores remained stable after 6 months of treatment compared with baseline; however, survivor bias may have influenced these findings, given the marked reduction in sample size [32] or absence of follow-up cognitive assessments [33]. Another study from Israel [38] reported a significant decline in MMSE scores over time among 53 patients who completed the 6-month assessment. Notably, a subgroup analysis revealed that this decline was significant only in younger participants (≤ 74 years), whereas no meaningful change was observed in older participants. Lastly, a US study [36], in a preliminary analysis conducted on the CDR-SB, showed that the estimated rate of change over the treatment period was 1.11 per year, which corresponded with the rate of change reported for the placebo group of CLARITY-AD [13].

#### Case reports

Seventeen case reports were identified from the literature search [39–55]. Further details of included studies are reported in Table S2. Fifteen studies described single case presentations [39–45, 48–55], while two studies described two cases each [46, 47] for a total of 19 individuals. Two studies reported evidence from two individuals included in a RCT [54, 55]. Ten subjects were females (52.6%), eight had a diagnosis of MCI due to AD, and 11 had at least one ε4 allele. The age of included individuals ranged from 57 to 82 years. When reported, the majority of patients had a history of comorbidities, especially hypertension and dyslipidaemia. Most of the studies reported an AE between the 3rd and 7th infusion, while in only two cases, AE occurred following the 13th infusion. The majority of reported AEs were ARIA (ARIA-E, isolated ARIA-H, or concomitant ARIA-E and ARIA-H). Additional serious AEs included one case of angina followed by pontine haemorrhage [41], two cases of seizures requiring hospitalisation [43, 55], and one ischemic stroke [54]. Some studies also described acute persistent urinary retention [44] and enlargement of a cavernous malformation [45]. Initial symptoms were heterogeneous and are detailed in Table S2. In several cases, imaging findings did not have corresponding clinical correlates. Most importantly, the two individuals included in the RCT subsequently died [54, 55]. In 11 studies, lecanemab was temporarily or permanently discontinued, while in only six studies, lecanemab was continued or resumed after temporary interruption.

### Post-marketing safety

**Fig. 1:**
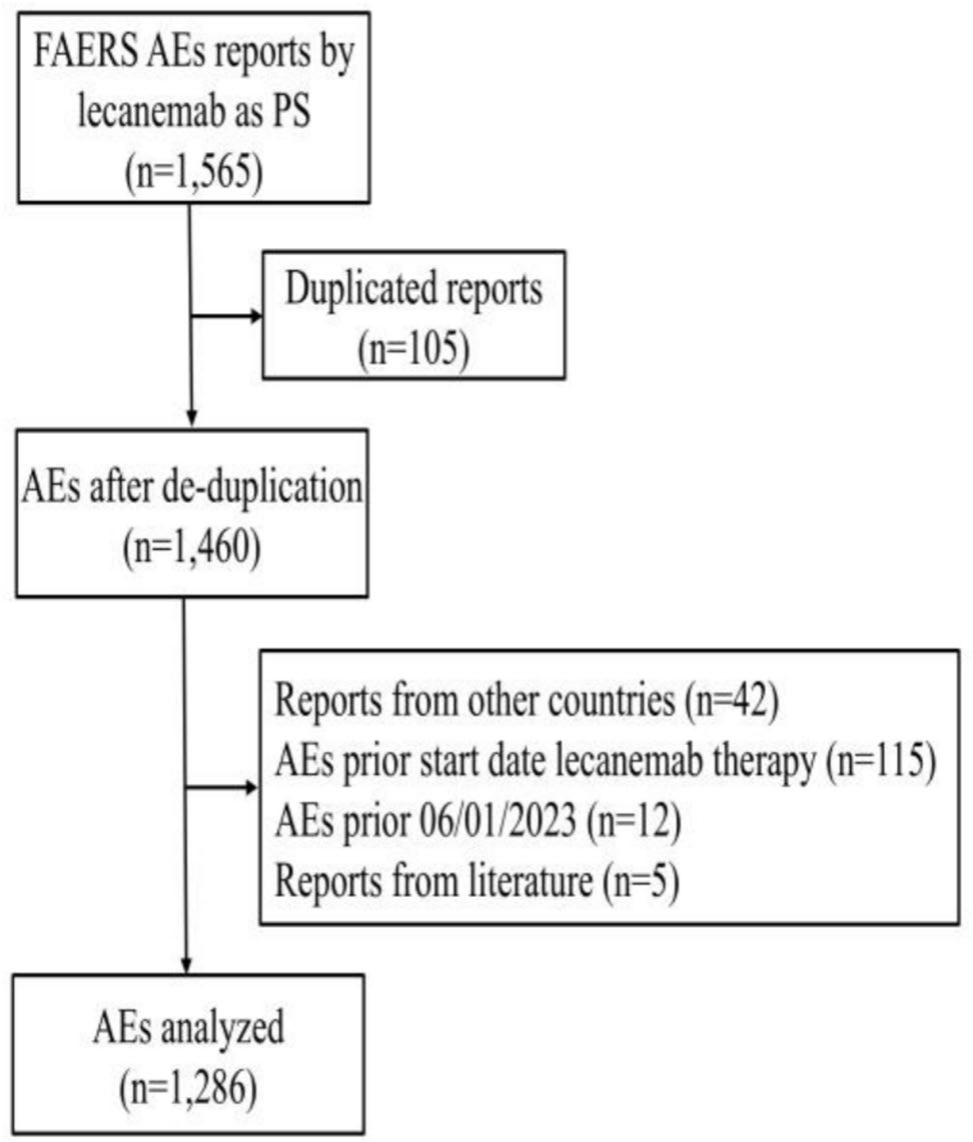
Flow-chart of the AEs reports selection from FAERS database. PS: Primary Suspect; AEs: Adverse Events

A total of 1,286 reports were analysed (Fig. 1). Eight reports involving treatment initiated before January 2023 were included. The analysis identified 2,627 adverse events (AEs), averaging 2.0 per report. About 88% of cases were reported in 2024, indicating increasing use and awareness of lecanemab. The US contributed 91.4% of reports (1,175), with healthcare professionals submitting 59.0%. Patient ages ranged from 37 to 92 years (mean = 73.6, SD = 7.4); 63.1% were over 65, and 51.6% were female. Missing data for age and gender were 28.1% and 11.4%, respectively. Lecanemab was indicated for “Alzheimer dementia” in 43.4% of reports and for “Cognitive disorder” in 5.2%.

Indication of use was not reported in almost half of the reports. About AEs severity, 385 reports (29.9%) concern SAEs, including 46 deaths corresponding to 3.6% of all AEs and 12% of SAEs. Moreover, other relevant serious outcomes were hospitalizations (15.2%), Important Medical Event (9.0%), disability (1.2%), and life-threatening (0.5%). The mean onset time, calculated from the start of therapy to the date of the AE, was 40 days (SD: 102 days, range: 0–1,283 days). It is opportune to remark that the AEs, including deaths, may be attributable to the underlying disease, concomitant medications or other causes not reported rather than lecanemab itself.

The most reported AEs were headache (n = 219, 8.3%), ARIA-E (n = 168, 6.4%), ARIA-H (n = 146, 5.6%), chills (n = 126, 4.8%), IRRs (n = 117, 4.5%), fatigue (n = 92, 3.5%), confusional state (n = 76, 2.9%), nausea (n = 75, 2.9%), pyrexia (n = 72, 2.7%), and dizziness (n = 65, 2.5%) (Table 2).

**Table 2:** Characteristics of the reports submitted to the US FDA Adverse Event Reporting System (FAERS) for lecanemab

Characteristics	No	%
**Total**	**1,286**	**100**
**Gender**		
Female	663	51.6
Male	477	37.1
Not specified	146	11.4
**Age, mean (SD)**	73.6 (7.4)	
18–44	1	0.1
45–64	112	8.7
65–74	347	27.0
75 +	464	36.1
Not specified	362	28.1
**Reporting year**		
2023	155	12.1
2024	1131	87.9
**Reporter**		
Consumer	495	38.5
Health professional (e.g. physician, pharmacist)	759	59.0
Not specified	32	2.5
Time to onset, days* lecanemab		
Total	722	56.1
Missing	564	43.9
Mean (SD)	40 (102)	
Median (Q1, Q3)	1 (0, 47)	
Min, Max	0, 1283	
**Indication of use**		
Dementia Alzheimer's	558	43.4
Cognitive disorder	67	5.2
Dementia	10	0.8
Other indications	2	0.2
Product used for unknown indication	331	25.7
Not specified	318	24.7
**Country**		
United States	1,175	91.4
Japan	111	8.6
**Severity**		
Non serious	901	70.1
Serious	385	29.9
**Reported outcome**		
Hospitalization	196	15.2
Other serious (Important Medical Event)	116	9.0
Death	46	3.6
Disability	16	1.2
Life-Threatening	7	0.5
Required intervention to prevent permanent impairment/damage	4	0.3
**Adverse events**	**2,627**	**100**
Headache	219	8.3
ARIA-E	168	6.4
ARIA-H	146	5.6
Chills	126	4.8
Infusion related reaction	117	4.5
Fatigue	92	3.5
Confusional state	76	2.9
Nausea	75	2.9
Pyrexia	72	2.7
Dizziness	65	2.5

^*^Time from the start of therapy to the date of Adverse Event

Forty-six deaths have been notified. More than 84% referred to the year 2024. Most of the deaths (about 85%) occurred in patients over 65 years, with one case involving a patient in the age category 45–64. The most frequently reported AEs were ARIA-E (n = 8, 17.4%), ARIA-H (n = 7, 15.2%), and cerebral haemorrhage (n = 5, 10.9%). Characterisation of deaths submitted to the FAERS is shown in Table S3 and in Table S4.

An analysis of ARIA related to lecanemab events submitted to the FAERS has been carried out. In most of the reports, ARIA-E (n = 168, 13.1%) and ARIA-H (n = 146, 11.4%) events have been notified. In 3.6% (n = 46) of cases, “ARIA” was generically reported. In 78 reports, both ARIA-E and ARIA-H events and in one report, ARIA-H and ARIA events were notified. All amyloid-related imaging abnormalities events (ARIAs) showed similar demographic characteristics in terms of gender (higher percentage of females), mean age ($\sim$73 years), reporting year (> 90% in 2024), reporter (healthcare professionals in > 76% of cases), and country (> 90% from the US). Data about severity showed a lower number of ARIAs reports with a high severity score (serious events in about 40% of cases). In particular, 21.4% of ARIA-E, 15.8% of ARIA-H, and 17.4% of ARIA cases required hospitalization; a similar number of deaths (8 vs 7 cases; 4.8% in both events) is reported for patients who experienced ARIA-E or ARIA-H events. Moreover, the median time to onset, defined as the time from the start of therapy to the date of the ARIAs, was very similar for each group (49 days for ARIA-E, 48 days for ARIA-H, and 43 days for ARIA). In general, this analysis showed more comparable data between ARIA-E and ARIA-H groups, while the ARIA group presents some differences (Table 3).

**Table 3:** Characterisation of ARIA events submitted to the US FDA Adverse Event Reporting System (FAERS) for lecanemab

**Characteristics**	**ARIA-E**		**ARIA-H**		ARIA*	
	**No**	**%**	**No**	**%**	**No**	**%**
**Total**	**168**	**13.1**	**146**	**11.4**	**46**	**3.6**
**Gender**						
Female	102	60.7	78	53.4	22	47.8
Male	47	28.0	51	34.9	6	13.0
Not specified	19	11.3	17	11.6	18	39.1
**Age, mean, SD**	72.8 (6,6)		73.6 (7.1)		72.8 (7.1)	
18–44	-	-	-	-	-	-
45–64	15	8.9	12	8.2	2	4.3
65–74	56	33.3	41	28.1	8	17.4
75 +	55	32.7	61	41.8	9	19.6
Not specified	42	25.0	32	21.9	27	58.7
**Reporting year**						
2023	7	4.2	8	5.5	2	4.3
2024	161	95.8	138	94.5	44	95.7
**Reporter**						
Consumer	29	17.3	28	19.2	9	19.6
Health professional (e.g. physician, pharmacist)	135	80.4	112	76.7	35	76.1
Not specified	4	2.4	6	4.1	2	4.3
**Country**						
United States	151	89.9	138	94.5	46	100.0
Japan	17	10.1	8	5.5	-	-
**Severity**						
Non serious	98	58.3	89	61.0	33	71.7
Serious	70	41.7	57	39.0	13	28.3
**Reported outcome**						
Hospitalization	36	21.4	23	15.8	8	17.4
Other serious (Important Medical Event)	18	10.7	20	13.7	4	8.7
Death	8	4.8	7	4.8	-	-
Disability	6	3.6	6	4.1	-	-
Life-Threatening	2	1.2	-	-	1	2.2
Required intervention to prevent permanent impairment/damage	-	-	1	0.7	-	-
Time to onset, days**						
Total	96	57.1	83	56.8	10	21.7
Missing	72	42.9	63	43.2	36	78.3
Median (IQR)	49 (37, 72)		48 (30, 74)		43 (34, 53)	

^*^ Not specified; ** Time from the start of therapy to the date of Adverse Event

## Discussion

This study used a mixed-method approach to evaluate the current RWE on lecanemab from three complementary standpoints: the regulatory, the clinical, and the pharmacovigilance perspective.

Evidence collected from product information underscores the variability in the lecanemab labels approved by four different regulatory authorities, particularly regarding treatment indications, patient selection, contraindications, discontinuation criteria, and ARIA event monitoring (Table 1). Despite differences in approval pathways, all four regulatory agencies indicate lecanemab for individuals with a clinical diagnosis of MCI or mild dementia due to AD with confirmation of amyloid pathology. However, the FDA and PMDA do not exclude ApoE ε4 homozygotes from receiving lecanemab, although a “black box” warning is included in the labels. Prescribers are advised to test for ApoE ε4 status before initiating treatment and to inform patients of the associated risk of developing ARIA. Before testing, prescribers should discuss with patients and caregivers the risk of ARIA across different genotypes and the potential implications of the results. In contrast, the EMA and MHRA exclude ApoE ε4 homozygotes from treatment. These differences in the eligible population should be carefully considered when comparing RWE from different geographic regions, including data collected from pharmacovigilance databases and pharmacoepidemiologic studies. Therefore, evaluating the external validity of the CLARITY-AD trial [13] is essential to establish the risk–benefit profile of lecanemab for its application in routine clinical practice across diverse healthcare systems. It is important to recognize that current real-world data largely originates from highly specialized centers, which are more likely to enroll patients in the earliest stages of disease. Evidence from non-academic institutions and less urbanized regions will therefore be essential to determine whether these trends are consistent across more diverse clinical settings. Among the included studies, only one [35] compared outcomes between an academic institution and a private clinical practice. As this evidence comes from a single study from the US, additional research is needed to evaluate how the use and impact of lecanemab may differ across centers with varying levels of expertise and organizational structures.

Our analysis of nine cohort studies revealed several notable differences in the characteristics of the enrolled populations. Although the CLARITY-AD trial included over 60% of participants with MCI, only four studies reported a predominance of MCI patients. Two studies from China enrolled participants with moderate-to-severe AD [31, 33]. Although the authors reported that patients and their caregivers were fully informed about potential risks, the use of lecanemab in this population remains off-label. Furthermore, there is no evidence supporting clinical efficacy in patients with dementia in advanced stages.

As expected, the included studies were conducted especially in countries where lecanemab was approved. However, most studies focused primarily on the safety and feasibility of lecanemab, while evidence on cognitive and functional outcomes remains limited. Studies assessing short-term clinical outcomes reported favourable results; nevertheless, these findings should be interpreted with caution due to short follow-up periods, high dropout rates, and the absence of control groups. Efficacy data from the pivotal trial demonstrated that 18 months of lecanemab was associated with a statistically significant less worsening in cognitive decline, as measured by the CDR-SB scale. Initial results underscore the need for more comprehensive and longer-term data to fully assess the clinical effectiveness of lecanemab [31–33, 36, 38]. Interestingly, clinical outcomes observed in the Chinese studies showed more favourable results than those reported in studies from the US and Israel [36, 38]. Data from the Asian regional cohort of the CLARITY-AD study may partially explain differences [56]. Indeed, the overall efficacy and biomarker changes observed in the Asian cohort were consistent with the results of the full trial population, while ARIA events and IRRs occurred less frequently than in the overall cohort. This finding warrants further investigation, as a lower incidence of AEs in Asian populations could allow for longer treatment duration and potentially greater clinical benefit. To date, evidence over three years of treatment across the core study and long-term extension of the CLARITY-AD showed that lecanemab reduced cognitive decline on the CDR-SB by 0.95 compared to the expected decline [57]. However, the lecanemab arm was not compared with an internal control group, but with an external historical cohort. This introduces major sources of bias, such as different inclusion/exclusion criteria and disease severity, unequal dropout rates, and different follow-up schedules.

From a public health perspective, it is essential to determine which patients achieve the minimal clinically important difference (MCID), indicating an improvement that is perceived as clinically meaningful. To address this, responder analyses are needed to quantify the proportion of patients treated with lecanemab who demonstrate a clinically relevant response. To date, only one study [31] has assessed the MCID for the ADAS-Cog14 as an outcome of interest. However, the results were reported solely as continuous data, with no responder proportion provided, and the analysis was further limited by a 50% reduction of the baseline cohort. Although evidence from cohort studies is stronger for determining the safety, feasibility, and effectiveness of lecanemab, seventeen case reports were included in this review as complementary evidence. The included case reports provide valuable insights into adverse events, highlighting novel safety issues that were not identified in RCTs. Among the included case reports, two deaths occurred. Both patients were ApoE homozygotes and they received three infusions before the event. In one case [55], clinical, pathological, and imaging findings were consistent with a severe ARIA. Neuropathology revealed marked perivascular inflammation and arteriolar damage. In the other case [54], multiple acute cerebral haemorrhages occurred. The patient developed many acute intracerebral haemorrhages after receiving intravenous t-PA for an acute ischemic stroke. This case suggests a potential association between the use of lecanemab in combination with thrombolytic agents and an increased risk of necrotizing vasculopathy and haemorrhagic complications. Furthermore, in most cases, lecanemab treatment was either temporarily or permanently discontinued. While such discontinuations may reflect a cautious approach during the early real-world use of lecanemab, they also highlight important considerations regarding treatment tolerability and management. Supporting this point, one of the included cohort studies [37], which followed 71 participants over 14 infusions (7 months), reported that only a single participant completed the full course, and less than 50% received more than five infusions.

ARIA remain the most common and closely monitored adverse events associated with lecanemab. Although the ε4 allele is linked to an increased risk of ARIA, RWE should be interpreted in the context of the prescribing information applicable in the specific region or country where the study is conducted. Excluding homozygotes may contribute to a lower incidence of AEs, particularly ARIA, in European cohorts.

Since its marketing approval, experts from various countries have convened to develop criteria for the appropriate use (AURs) of lecanemab in clinical practice [58–60]. While the drug labels remain the only legally binding documents, AURs serve as additional support tools for prescribers. Despite the central role of the CLARITY-AD criteria in shaping regulatory labels, the various AURs developed internationally show considerable heterogeneity. Because different expert groups produced their own guidance, the resulting recommendations diverge on several key points. For instance, although the CLARITY-AD trial applied age-based exclusion criteria, all AURs consistently advise against imposing age limits in clinical practice. In contrast, the AURs differ in how they operationalize cognitive and functional assessments to determine patient eligibility, as well as in whether individuals with atypical Alzheimer’s disease phenotypes should be considered candidates for treatment. Interestingly, only the French AURs considered frailty as a screening tools to refer older patients for a comprehensive geriatric assessment before starting therapy [59]. Moreover, it is important to emphasize that the MMSE does not adequately capture the full spectrum of cognitive functions necessary to determine treatment eligibility. As highlighted in the AURs, several factors can influence MMSE performance and should be considered, including progressive aphasia, low educational level, and non-native language proficiency. Additional discrepancies include the management of genetic risk: the French AURs recommend excluding ApoEε4 homozygotes, whereas US AURs, in accordance with the FDA label, recommend only extreme caution in this population. Variations are also evident in MRI monitoring protocols for ARIA and in the handling of concomitant therapies, particularly anticoagulant use. A detailed comparison of these differences is provided in Table S6. Therefore, these factors should be considered in the context of the heterogeneity of real-world populations.

In the FAERS database, the reported indications for lecanemab use are highly heterogeneous (Table 2), preventing a clear identification of the patients' diagnostic profiles. According to FAERS, 51.6% of reported cases were female, and 36.1% were aged over 75. However, FAERS does not collect information on ApoE genotype or the methods used to confirm amyloid pathology. One in three of AEs were classified as serious, and among these, half required hospitalization; among all ARIA cases, 17.4% required hospitalization. A total of 46 deaths were reported, and of these, 15 patients had experienced ARIA-E or ARIA-H events, and 5 had suffered cerebral haemorrhage. Moreover, as previous studies aimed to evaluate the safety of lecanemab in the post-marketing period by assessing the reports submitted to the FAERS, this study reported some safety concerns associated with lecanemab to ARIA, and neurological events in addition to frequently notified AEs such as IRR, headache, fever, and fatigue [16–22]. The FAERS database includes AE reports submitted to the FDA by manufacturers, as mandated, as well as spontaneous reports from healthcare professionals and patients. Importantly, as explicitly stated by the FDA, FAERS data do not establish causal relationships between a drug and reported outcomes. Moreover, the FDA advises that FAERS data should not be used to determine the incidence of AEs in treated populations [28]. Therefore, such data should be interpreted not as definitive indicators of a drug’s safety profile, but as potential safety signals requiring further evaluation [61]. Pharmacovigilance systems provide safety signals that require further investigation through pharmacoepidemiologic studies. Pharmacovigilance and pharmacoepidemiology should operate synergistically to strengthen post-marketing safety surveillance [62]. An important aspect to consider is equity in access to treatment. The EMA has excluded homozygotes for the ε4 allele from receiving the therapy, those with the highest risk of developing ARIA. While this exclusion increases the safety profile of the treatment, it simultaneously affects a subgroup with a higher likelihood of developing AD. Moreover, differences in healthcare models across countries (e.g., insurance-based systems versus universal coverage) will influence equity in access to high-cost therapies such as lecanemab. In some settings, certain procedures may require out-of-pocket payment, limiting access to individuals with higher socioeconomic resources. Furthermore, in some national healthcare systems, lecanemab is not currently reimbursed with clear implications for equity in access to treatment [9, 10].

Patient registries are expected to become a cornerstone of post-marketing surveillance for ATT such as lecanemab and donanemab. In the US, this framework is already exemplified by the ALZ-NET registry, while the EMA and MHRA are moving toward controlled-access programs designed to ensure additional measures of risk minimisation. Yet the value of registries should extend beyond safety monitoring alone. Well-designed registries are essential for generating high-quality evidence on real-world effectiveness, treatment patterns, and long-term outcomes. Japan offers an instructive model, where a national registry supported through a public–private partnership aims to promote rigorous data collection, address potential conflicts of interest, and enable continuous evaluation of the risk–benefit profiles [63]. Such initiatives ultimately serve a single purpose: safeguarding patient safety while optimizing the impact of ATT.

This study has several strengths. The analysis encompasses three distinct domains (regulatory, clinical, and post-marketing), allowing for a comprehensive overview of real-world evidence on lecanemab. This multidimensionality provides valuable guidance for clinicians regarding patient management. Comparing the prescribing information from four regulatory authorities offered insight into the international evaluation of lecanemab (including differences in the approach to the emerging paradigm of biological diagnosis in AD). Moreover, the evidence concerning the use of lecanemab in clinical practice represents a novel contribution. Lastly, the inclusion of pharmacovigilance data was considered valuable, as it serves as a useful monitoring tool for observing many individuals treated in uncontrolled clinical settings – unlike the controlled environment of RCT. Furthermore, applying geographical restrictions to the FAERS dataset helped ensure greater consistency by focusing on regions with a longer history of lecanemab use, rather than including data from countries where the drug was only recently introduced into clinical practice.

However, this study has some limitations. The decision to include only a subset of regulatory authorities represents a limitation, as other agencies could have provided additional insights. We are aware of the limited number of SmPCs considered, but we do not expect substantial differences or additional information provided by other regulatory agencies beyond that provided by the influential agencies included in the study. Moreover, the literature review was conducted using only two databases. These databases were chosen as primary sources because they provide broad, complementary, and high-quality coverage of biomedical and pharmacological research, ensuring a robust and comprehensive identification of relevant studies. While relying on a limited number of databases may have excluded some publications, their selection supports the rigor and reliability of the evidence captured. Evidence from the literature was based primarily on data derived from cohort studies with a limited number of participants and relatively short follow-up periods, as well as from individual case reports. These factors may affect the generalizability and robustness of the conclusions drawn. Last, FAERS database present some limitations: i) it relies on voluntary reporting, which may lead to selection bias, underreporting, and incomplete data; ii) it lacks detailed clinical background information about patients, such as medical history, genetic risk factor for AD, and concomitant medications; iii) rates of occurrence cannot be calculated due to the absence of data on use (e.g. number of treated patients, number of used packages, etc.); iv) it doesn’t allow to classify the likelihood of a causal relationship between drug and AE. The AEs, including deaths, may be attributable to the underlying disease or concomitant medications rather than lecanemab itself. Therefore, clinical conclusions and treatment recommendations cannot be extrapolated from the FAERS database analyses [62, 64]. Considering the above-mentioned limitations and low quality of evidence from data about SAEs as reported in pharmacovigilance databases, further controlled clinical trials and large-scale cohort studies are needed to robustly assess the risk/benefit ratio of lecanemab.

## Conclusions

This study underscores the complexity of lecanemab’s safety profile and the variability in prescribing information issued by different regulatory authorities. Although there is a consensus regarding the risk of ARIA – particularly among individuals homozygous for the ApoE ε4 allele – recommendations for clinical management differ markedly. Evidence from real-world sources identifies ARIA as the most commonly reported adverse event, typically presenting in mild forms but occasionally resulting in severe or fatal outcomes. These findings highlight the critical need for continued post-marketing surveillance and the development of harmonized recommendations. The generation of robust, high-quality real-world evidence will be essential to inform policy decisions and ensure the safe and effective integration of lecanemab into routine clinical practice.

## Supplementary Information

Below is the link to the electronic supplementary material.

Supplementary file1 (DOCX 105 KB)

Supplementary file2 (PDF 209 KB)

## Data Availability

The datasets analyzed in this study are available in the free, open-access FDA Adverse Event Reporting System (FAERS) repository available at: [https://www.fda.gov/drugs/questions-and-answers-fdas-adverse-event-reporting-systemfaers/fda-adverse-event-reporting-system-faers-public-dashboard].

## Declarations

### Disclaimers

The views expressed in this article are the personal views of the authors and may not be understood or quoted as being made on behalf of or reflecting the position of the respective authors’ organisations.
