# Supplementary material for: Regulatory, clinical, and post-marketing challenges of lecanemab for Alzheimer’s disease: insights from real-world data
Source: Neurol Sci. 2026 Mar 6;47(4):322. doi: 10.1007/s10072-026-08829-4 (PMC12963267; doi:10.1007/s10072-026-08829-4)
Supplement: Supplementary file 1 — Supplementary file1 (DOCX 105 KB) [file 10072_2026_8829_MOESM1_ESM.docx]

**Regulatory, clinical, and post-marketing challenges of lecanemab for Alzheimer’s disease: Insights from real-world data**

**Supplementary materials**

**Figure S1.** PRISMA flow diagram of included studies (literature updated to November 16, 2025)

**Table S1.** Case presentations described in the included cohort studies

**Table S2.** Case presentation, outcomes and treatment status in the included case reports

**Table S3.** Characterisation of deaths for Adverse Event Reports submitted to the US FDA Adverse Event Reporting System (FAERS) for lecanemab

**Table S4.** Main characteristics of cases of deaths for all causes in patients treated with lecanemab submitted to the US FDA Adverse Event Reporting System (FAERS).

**Table S5.** Comparisons between the CLARITY-AD trial and cohort studies from real-world

**Table S6.** Comparisons between the CLARITY-AD trial and experts Appropriate Use Recommendations (AURs)

**Table S7.** Checklist for prevalence/incidence studies (cohort studies)

**Table S8.** Checklist for case reports

**Table S1.** Case presentations described in the included cohort studies

| **Reference** | **Country** | **Case(s) presentation** | | | | | | **Outcome(s)** | **Treatment status** | |
| --- | --- | --- | --- | --- | --- | --- | --- | --- | --- | --- |
|  |  | **Demographic and clinical presentation** | | **ARIA event(s)** | | **Clinical symptoms** | |  |  |  |
| **Paczynski**  **2025 [36]** | US | F, ~70, AD, ApoE ε3/ε3 | | Moderate ARIA-E and ARIA-H with mild MH and severe superficial siderosis (4^th^ infusion) | | Mild headache. Then, subacute worsening of cognition and psychosis, right upper-extremity weakness, and right homonymous hemianopsia | | Headaches, visual field deficit, weakness, and psychosis improved.  Hospitalized and treated with high-dose intravenous steroids for 5 days; discharged on an oral steroid taper  Still required ongoing treatment with antipsychotics. | Lecanemab infusions held until symptoms resolved. | |
|  |  | M, ~65, AD, ApoE ε3/ε3 | | Moderate ARIA-E and ARIA-H with moderate MH  (4^th^ infusion) | | Mild headache, acute worsening of cognition, gait disturbance | | Hospitalized and treated with high-dose intravenous steroids for 5 days; discharged on an oral steroid taper. MRI at 3 months showed resolving ARIA-E and stable ARIA-H. Symptoms resolved and neurological examination returned to pretreatment baseline. | Lecanemab infusions held until symptoms resolved. | |
|  |  | F, ~80, AD, ApoE ε3/ε4 | | Moderate ARIA-E and ARIA-H with severe MH and severe superficial siderosis (4^th^ infusion) | | Headache, subacute cognitive decline, aphasia, visual disturbance, right arm tremor, shuffling gait | | MRI at follow-up showed resolving ARIA-E and stable ARIA-H; continued improvement in aphasia and confusion. | Lecanemab infusions held until symptoms resolved. | |
|  |  | M, ~70, AD, ApoE ε4/ε4 | | Moderate ARIA-E and ARIA-H with mild MH (4^th^ infusion) | | Headache, subacute decline in cognition, agitation | | Follow-up MRI 2 showed improving ARIA-E, 1 new MCH; symptoms resolved. | Lecanemab infusions held until symptoms resolved. | |
|  |  | F, ~80, MCI due to AD, ApoE ε4/ε4 | | Moderate ARIA-E and ARIA-H with severe MH (4^th^ infusion) | | Subacute decline in cognition | | Follow-up MRI 2 showed resolving ARIA-E and stable ARIA-H; symptoms resolved. | Lecanemab infusions held until symptoms resolved. | |
|  |  | F, ~75, AD, ApoE ε3/ε4 | | Moderate ARIA-E and ARIA-H with moderate MH (4^th^ infusion) | | Change in visual acuity and colour perception | | Follow-up MRI 3 showed resolving ARIA-E and stabilize ARIA-H; symptoms resolved. | Lecanemab infusions held until symptoms resolved. | |
|  |  | F, ~70, MCI due to AD, ApoE ε4/ε4 | | Severe ARIA-E and ARIA-H with moderate MH and mild superficial siderosis (4^th^ infusion) | | Headache, new onset bilateral upper extremity tremor | | Follow-up MRI 4 showed improving ARIA-E and stable ARIA-H; symptoms resolved. | Lecanemab infusions held until symptoms resolved. | |
|  |  | F, ~60, AD, ApoE ε3/ε4 | | Moderate ARIA-E and ARIA-H with severe MH (4^th^ infusion) | | Headache | | Follow-up MRI 4 showed nearly resolved ARIA-E and stable ARIA-H; ongoing management of mild headaches. | Lecanemab infusions held until symptoms resolved. | |
|  |  | F, ~80, MCI due to AD, ApoE ε2/ε3 | | Moderate ARIA-E and ARIA-H with mild MH (4^th^ infusion) | | Headache, dizziness, visual disturbance, agitation | | Follow-up MRI 1 showed resolved ARIA-E and stable ARIA-H; symptoms resolved. | Lecanemab infusions held until symptoms resolved. | |
|  |  | F, ~60, AD, ApoE ε3/ε4 | | Mild ARIA-E and ARIA-H with mild MH (4^th^ infusion) | | Headache | | Follow-up MRI 2 showed resolved ARIA-E and stable ARIA-H; symptoms resolved. | Lecanemab infusions held until symptoms resolved. | |
|  |  | M, ~65, AD, ApoE ε3/ε4 | | Mild ARIA-E and ARIA-H with mild MH (13^th^ infusion) | | Visual disturbance | | Follow-up MRI 2 showed resolved ARIA-E and stable ARIA-H; symptoms resolved. | Lecanemab infusions held until symptoms resolved. | |
| **Shields**  **2024 [37]** | US | M, 67, ApoE ε4/ε4, hypertension,  hypercholesterolemia, diabetes mellitus | | Moderate ARIA-H (before 5^th^ infusion) | | Asymptomatic | | 1st MRI following ARIA negative for new ARIA- H and ARIA-E | Lecanemab temporarily interrupted | |
|  |  | M, 80, ApoE ε3/ε4, hypertension  hypercholesterolemia, diabetes mellitus, cerebrovascular disease | | Moderate ARIA-H (before 5^th^ infusion) | | Symptomatic | | n/r | Lecanemab permanently discontinued | |
|  |  | M, 72, ApoE ε3/ε4, hypertension  hypercholesterolemia, diabetes mellitus, cerebrovascular disease | | Mild  ARIA-E | | Asymptomatic | | 1^st^ MRI following ARIA negative for new ARIA-H and ARIA-E. Previous ARIA-E resolved | Lecanemab continued | |
|  |  | F, 77, ApoE ε3/ε3, hypertension | | Mild ARIA-H and ARIA-E | | Asymptomatic | | n/r | Lecanemab continued | |
|  |  | M, 74, ApoE ε3/ε3  hypercholesterolemia | | Mild  ARIA-E (before 5^th^ infusion) | | Asymptomatic | | n/r | Lecanemab continued | |
|  |  | F, 71, ApoE ε4/ε4 | | Moderate ARIA-H and ARIA-E (before 5^th^ infusion) | | Headaches | | n/r | Lecanemab permanently discontinued | |
|  |  | F,71, ApoE ε3/ε4  hypercholesterolemia | | Mild  ARIA-E (before 5^th^ infusion) | | Headaches | | n/r | Lecanemab permanently discontinued | |
|  |  | F, 75, ApoE ε4/ε4  hypertension, hypercholesterolemia | | Mild ARIA-H (before 5^th^ infusion) | | Asymptomatic | | 1^st^ MRI following ARIA negative for new ARIA-H and ARIA-E | Lecanemab continued | |
|  |  | F, 74, ApoE ε3/ε4  hypertension, hypercholesterolemia | | Mild ARIA-H (before 5^th^ infusion) | | Asymptomatic | | ARIA-E increased in size | Lecanemab was permanently discontinued | |
|  |  | F, 73, ApoE ε4/ε4  hypercholesterolemia | | Mild ARIA-E and  mild ARIA-H  (before 5^th^ infusion) | | Disorientation for several days | | Increased size of ARIA-E and new foci of ARIA-H | Lecanemab was permanently discontinued | |
|  |  | F, 71, ApoE ε2/ε4  hypercholesterolemia | | Mild ARIA-H  (before 5^th^ infusion) | | Asymptomatic | | n/r | Lecanemab continued | |
| **Arai 2025 [30]** | **Japan** | No disaggregated data available | | | | | | | | |
| **Bregman 2025 [38]** | **Israel** | No disaggregated data available | | | | | | | | |
| **Chen 2025 [31]** | **China** | No disaggregated data available | | | | | | | | |
| **Kang 2025 [32]** | **China** | No disaggregated data available | | | | | | | | |
| **Li 2025 [33]*** | **China** | F, 60, ApoE ε3/ε4 | Mild ARIA-H  (before 14^th^ infusion) | | Chest distress | | MRI at follow-up identified 4 new microbleeds | | | Lecanemab was permanently discontinued |
|  |  | F, 73, ApoE ε4/ε4 | Moderate ARIA-E and severe ARIA-H | | Asymptomatic | | ARIA-E resolved after discontinuation | | | Lecanemab was permanently discontinued |
|  |  | F, 56, ApoE ε3/ε4 | - | | Headache | | No ARIA occurred | | | Lecanemab was self- discontinued by participant |
|  |  | F, 80, ApoE ε3/ε4 | Mild ARIA-H | | Dizziness and vomiting | | n/r | | | Lecanemab was permanently discontinued |
|  |  | M, 65, ApoE ε3/ε3 | Mild ARIA-E and moderate ARIA-H | | Asymptomatic | | ARIA-E resolved after 2 months | | | Lecanemab was permanently discontinued |
|  |  | F, 74, ApoE ε3/ε3 |  | | Fever, chills, and fatigue due to infusion-related reaction (1^st^ infusion) | | n/r | | | Lecanemab was permanently discontinued |
|  |  | F, 58, ApoE n/r | Moderate ARIA-H | | Dizziness with  eyelid apraxia, gait  instability,  photophobia,  interrupted sleep | | Symptoms partially resolved after 2 weeks of treatment interruption | | | Lecanemab was permanently discontinued |
|  |  | F, 79, ApoE ε2/ε4 | Moderate ARIA-H (after 4^th^ infusion) | | Movement disorder and incontinence | | Patient’s symptoms relieved after hormone therapy | | | Lecanemab was permanently discontinued |
|  |  | M, 61, ApoE n/r |  | | Allergies due to infusion-related reaction (1^st^ infusion) | | n/r | | | Lecanemab was permanently discontinued |
|  |  | F, 48, ApoE ε3/ε3 | Moderate ARIA-E and ARIA-H | | Asymptomatic | | n/r | | | Lecanemab was self- discontinued by participant |
|  |  | F, 47, ApoE ε3/ε4 | - | | Venous thrombosis of the lower extremities (after 11^th^ infusion) | | Deep vein thrombosis treated with rivaroxaban and partial recanalization chieved after 3 months | | | Lecanemab was permanently discontinued |
|  |  | F, 75, ApoE ε3/ε3 | - | | Atony, fatigue, lethargy, diarrhea and paranoid delusions against family members (after 1^st^ infusion) | |  | | | Lecanemab was permanently discontinued (after 4^th^ infusion) |
|  |  | F, 82, ApoE ε3/ε4 | - | | Hallucination (4^th^ infusion) | | n/r | | | Lecanemab was permanently discontinued (after 4^th^ infusion) |
|  |  | F, 74, ApoE ε4/ε4 | Severe ARIA-E and mild ARIA-H | | Slow in reacting | | The edema improved in the second MRI and the patient's symptoms  relieved after hormone therapy | | | Lecanemab was permanently discontinued (after 5^th^ infusion) due to symptomatic ARIA-E |
|  |  | M, 72, ApoE ε3/ε3 | Mild ARIA-H (before 5^th^ infusion) | | Profuse sweating,  vomiting, fatigue | | n/r | | | Lecanemab was permanently discontinued (after 5^th^ infusion) due to IRR and ARIA-H |
|  |  | M, 64, ApoE n/r | n/r | | Fever and chills due to IRR occurred after the 1^st^ infusion | | n/r | | | Lecanemab was self- discontinued by participant |
|  |  | F, 67, ApoE n/r | n/r | | Pain in the arm and  inability to lift | | Symptoms resolved spontaneously after the 5^th^ infusion | | | Lecanemab was permanently discontinued |
|  |  | M, 64, ApoE ε3/ε4 | Mild ARIA-E and mild ARIA-H | | Weakness in lower  limbs | | ARIA-E resolved after discontinuation | | | Lecanemab was permanently discontinued |
|  |  | F, 66, ApoE ε3/ε4 | Severe ARIA-H (before 8^th^ infusion) | | Asymptomatic | | Subsequent clinical vigilance and re-examination | | | Lecanemab was permanently discontinued |
|  |  | M, 82, ApoE ε3/ε3 | Severe ARIA-H (before 14^th^ infusion) 14mm hemorrhage | | Asymptomatic | | n/r | | | Lecanemab was permanently discontinued due to ARIA-H finding |
|  |  | F, 78, ApoE ε3/ε3 | Mild ARIA-E and severe ARIA-H | | Fever | | Mild ARIA-H was reported on the first and second MRIs, treatment continued.  The third MRI revealed severe  ARIA-H with 16 newly discovered  mH, as well as mild ARIA-E. | | | Lecanemab was permanently discontinued due to ARIA events |
| **Mervosh 2025 [34]** | **US** | No disaggregated data available | | | | | | | | |
| **Rosenbloom 2025 [35]** | **US** | No disaggregated data available | | | | | | | | |

* In the study by Li et al., the case descriptions referred exclusively to participants who discontinued treatment due to an adverse event.

AD: Alzheimer’s disease; ARIA-E: Amyloid-related imaging abnormalities – edema; ApoE: Apoliprotein E; ARIA-H: Amyloid-related imaging abnormalities – hemorrhage; CSF: Cerebrospinal fluid; ED: emergency department; EEG: electroencephalogram; EO: early-onset; F: Female; LO: late-onset; M: Male; MRI: Magnetic resonance imaging; MHs: microherrohages.

**Table S2.** Case presentation, outcomes and treatment status in the included case reports

| **Reference** | **Country** | **Case(s) presentation** | | | **Outcome(s)** | **Treatment status** |
| --- | --- | --- | --- | --- | --- | --- |
|  |  | **Demographic and clinical presentation** | **Adverse event(s)** | **Initial symptoms** |  |  |
| **Alammar 2025 [39]** | US | F, 76, MCI due to AD, ApoE ε2/ε3  Without typical stroke risk factors and a history of bladder cancer | Evidence of 7mm hemosiderin deposition consistent with ARIA-H (4^th^ infusion); Evidence of mild ARIA-E (6^th^ infusion) | Asymptomatic. Slightly worsened memory and experience of agitation at night (reported by proxy) | On the MRI surveillance scan, several ischemic strokes were observed. The patient underwent a stroke workup that was negative, even after 13 days of cardiac monitoring | Lecanemab was stopped |
| **Bitar 2025 [40]** | US | F, 60, mild AD  ApoE n/r | Cerebral edema consistent with ARIA-E (6^th^ infusion) | Asymptomatic | The patient remained stable without  new neurologic deficits | Lecanemab was stopped conservatively |
| **Chen 2025 [41]** | China/US | F, 76, MCI due to AD, ApoE ε3/ε3  coronary artery  disease, DVT, stable breast cancer; dual antithrombotic therapy before lecanemab | Due to angina, the patient underwent coronary stenting. Occurrence of pontine hemorrhage 20 days after stenting | Sudden dizziness with dyplopia | Antithrombotic therapy was adjusted, and rehabilitation treatment; the patient’s ICH was gradually absorbed, but intermittent dizziness remained. Physical examination still showed mild abduction limitation of the left eye, but  no obvious diplopia symptoms | Lecanemab was paused at the time of recurrent angina. *Probably not resumed*. |
| **Hanyu 2025 [42]** | Japan | M, 75, cognitive decline consistent with mild dementia (CDR=0.5) ApoE ε4/ε4 | None | None | Follow-up MRI displayed no ARIA or other adverse findings. Cognition improved on CDR-SB; MMSE and MoCA showed no changes. Amyloid clearance after treatment (51.6 to 3.6 centiloid) | The patient received lecanemab until 18 months. *Not clearly reported treatment after 18 months.* |
| **Naeshiro 2025 [43]** | Japan | M, 75, MCI due to AD, ApoE ε3/ε4; history of myocardial infarction,  hypertension, and dyslipidemia | Generalized seizures after the 3^rd^ infusion. MRI findings showed microinfarctions and evidence of severe ARIA-E | Mild concentration difficulties preceding seizures | High-dose intravenous methylprednisolone and midazolam for seizure control. When seizures had resolved, midazolam was discontinued, and consciousness had recovered.  Oral prednisolone, levetiracetam, and lacosamide were initiated.  Prednisolone was tapered and discontinued after one month. MRI abnormalities gradually resolved, with a complete resolution (day 80).  CSF Aβ40 levels transiently decreased by 30% during the ARIA episode, whereas Aβ42 remained stable, thereby  increasing the Aβ42/40 ratio | Lecanemab was resumed after 174 days of interruption |
| **Navarra 2025 [44]** | US | M, 65, mild AD, ApoE ε3/ε4; history of BPH, 10-year history of SIADH and history of ADHD | Acute persistent urinary retention (AUR) following two consecutive lecanemab infusions | - | After 2 days of persistence, at the ED the patient received a catheterization. A catheter was recommended to stay in for 2 weeks. Due to discomfort, the catheter was removed after 5 days.  After the 2nd infusion the patient had difficulty voiding and was again diagnosed with AUR. Catheterization released over 1L of urine, and a urinary catheter was recommended to stay in for 2 weeks. | Lecanemab was stopped after the second infusion due to concern that it was the trigger for urinary retention.  *The Naranjo score was calculated and the score was 6* |
| **Schwartz 2025 [45]** |  | F, 73, MCI due to AD, ApoE ε3/ε4; history of hypertension, hyperlipidemia, carcinoma. Incidental identification of cerebral cavernous malformation | Surveillance MRI demonstrated enlargement of cavernous malformation (13mm) with development of subacute blood  products but no edema or acute hemorrhage (13^th^ infusion) | No particular symptoms occurred | Follow-up MRI showed reduction in the cavernous malformation. Amyloid PET-CT after 13^th^ infusion was positive with Centiloid value of 53, suggesting incomplete clearance. No baseline PET was available. There was no substantial change in the patient’s adaptive function over treatment | Lecanemab was stopped giving the increasing risk of hemorrhage |
| **Wang Y. 2025 [46]** | China | **Case 1.** M, 70, AD, ApoE ε2/ε3; history of hypertension | Mild ARIA-E and ARIA-H (5^th^ infusion) | Asymptomatic at the time of the 5^th^ infusion | Before the 7^th^ infusion, the patient presented mild dizziness and noticeable decline in memory, reduced comprehension, executive function, and cognitive abilities reported by proxy. Significant increase in mH and progression of ARIA-E | Lecanemab was stopped due to elevated risk of cerebrovascular events |
|  |  | **Case 2.** F, 73, MCI due to AD, ApoE ε3/ε4; history of hypertension | Mild ARIA-H (7^th^ infusion) | Asymptomatic | No particular descriptions. Close monitoring in clinical status | Lecanemab was continued |
| **Wang X. 2025 [47]** | China | **Case 1.** F, 67, moderate AD (MMSE 12/30), ApoE ε3/ε4; sleep disturbances, depression | - | - | Following 4^th^ doses of lecanemab,  stability in cognitive function,  worsening in daily functioning  increased caregiver burden, and secondary suicidal ideation.  It prompted the initiation of psychiatric interventions. Then, patient’s mood improved, with stabilization achieved within approximately 2 weeks and concomitant improvement in sleep | Lecanemab was continued |
|  |  | **Case 2.** M, 73, mild AD, ApoE ε3/ε4 | - | - | - | Lecanemab was continued |
| **Watanabe 2025 [49]** | Japan | F, 70, MCI due to AD, ApoE ε3/ε3  hypertension | ARIA-H accompanied by concomitant spontaneous hemorrhage in the pre-existing subdural hygroma (3^rd^ infusion) | loss of appetite | Cognitive decline and mild verbal paraphasia and difficulty with word recall in free conversation  No other major neurological deficits | Lecanemab was stopped after the diagnosis of intracranial hemorrhage |
| **Zou 2025 [48]** | China | M, 82, AD, ApoE ε3/ε3 | Markedly increase in D-dimer levels (>20 µg/mL) | Dizziness, fatigue, and palpitations | To prevent other potential thromboembolic events, the patient received one day treatment with rivaroxaban (10mg) and his D-dimer  level returned to 6.4 µg/mL. Due  to concerns about potential additional AEs, lecanemab was adjusted to 200 mg | Lecanemab was continued |
| **Gibson 2024 [50]** | US | M, 71, Mild dementia, ApoE ε3/ε4,  mild hypertension | Severe ARIA-E with mild ARIA-H | Gradual onset bifrontal headache after the third infusion; episode of confusion lasting approximately 30 minutes; severe headache prompting ED presentation | Patient alive 6 months after lecanemab discontinuation; CSF evaluation showed AD biomarkers improvement after only 3 doses | Lecanemab was stopped after the third infusion |
| **Ji 2024 [51]** | US | M, 73, A+/T+/N+ AD diagnosis  MMSE 29/30,  ApoE ε2/ε3  hypertension  dyslipidemia, Type II diabetes | ARIA-H (3 new MHs during a surveillance MRI following the 13^th^ infusion) | Head laceration due to fall (no loss of consciousness) | Patient alive 1 month later, largely asymptomatic, drop in the MMSE score to 26 (losing points for verbal recall and serial 7s compared to initial exam). | Lecanemab was stopped |
| **Noguchi-Shinohara 2024 [52]** | Japan | F, 69, aMCI,  MMSE 27/30,  ApoE ε4/ε4  Comorbidities n/r | Moderate ARIA-E (before 5^th^ infusion) | Asymptomatic | Hospital admission and treatment with intravenous methylprednisolone 1g/day for 3 days and then prednisolone 20mg/day for 2 weeks, gradually tapered and discontinued after 1 month. MRI at 1 month showed resolution of ARIA. | n/r |
| **Yamazaki**  **2024 [53]** | Japan | F, 57, EO-AD, MMSE 26/30, ApoE n/r | Severe ARIA-E with ARIA-H (4^th^ infusion) | Left hemiparesis, confusion, headache, urinary incontinence | Patient alive at 40^th^ day of illness. MMSE and MoCA were 14/30 (-12 from baseline) and 11/30 respectively (-7 from baseline) | n/r |
| **Reish**  **2023* [54]** | US | Sex: n/r, 65, early stage of cognitive decline,  ApoE ε4/ε4 | Ischemic stroke  (3^rd^ infusion) | Acute onset of aphasia and left gaze preference MRI performed 81 days before the stroke showed mild small-vessel disease, with no microhemorrhages, edema, or ARIA | The patients received comfort measures and subsequently died | Lecanemab stopped |
| **Solopova 2023* [55]** | US | F, 79, mild AD,  ApoE ε4/ε4 | Suspected ARIA, seizure on hospital admission  (3^rd^ infusion) | Worsening cognitive decline, brain fog | The patient died 5 days after hospital admission due to aspiration event leading to sepsis with multiorgan failure  Neuropathological findings showed acute arteritis | Lecanemab stopped |

*Included in a randomized controlled trial

AD: Alzheimer’s disease; ADHD: Attention-Deficit Hyperactivity Disorder; ARIA-E: Amyloid-related imaging abnormalities – edema; ApoE: Apoliprotein E; ARIA-H: Amyloid-related imaging abnormalities – hemorrhage; CSF: Cerebrospinal fluid; DVT: deep vein thrombosis; ED: emergency department; EEG: electroencephalogram; EO: early-onset; F: Female; LO: late-onset; M: Male; MRI: Magnetic resonance imaging; MHs: microherrohages; SIADH: syndrome of inappropriate antidiuretic hormone secretion

**Table S3.** Characterisation of deaths for Adverse Event Reports submitted to the US FDA Adverse Event Reporting System (FAERS) for lecanemab

| **Characteristics** | **No** | **%** |
| --- | --- | --- |
| **Total** | **46** | **100** |
| **Gender** |  | |
| Female | 24 | 52.2 |
| Male | 20 | 43.5 |
| Not specified | 2 | 4.3 |
| **Age, mean (SD)** | 76.8 (5.4) |  |
| 45-64 | 1 | 2.2 |
| 65-74 | 12 | 26.1 |
| 75+ | 27 | 58.7 |
| Not specified | 6 | 13.0 |
| **Reporting year** | | |
| 2023 | 7 | 15.2 |
| 2024 | 39 | 84.8 |
| **Reporter** | | |
| Consumer | 4 | 8.7 |
| Health professional (e.g. physician, pharmacist) | 38 | 82.6 |
| Not specified | 4 | 8.7 |
| **Adverse events** | | |
| ARIA-E | 8 | 17.4 |
| ARIA-H | 7 | 15.2 |
| Cerebral hemorrhage | 5 | 10.9 |
| Nausea | 4 | 8.7 |
| **Time to onset, days* lecanemab** | | |
| Total | 31 | 67.4 |
| Missing | 15 | 32.6 |
| Mean (SD) | 270 (356) | |
| Median (Q1, Q3) | 132 (46, 264) | |
| Min, Max | 0, 1283 | |
| **Indication of use** | | |
| Dementia Alzheimer's type | 21 | 45.7 |
| Cognitive disorder | 1 | 2.2 |
| Product used for unknown indication | 2 | 4.3 |
| Not specified | 22 | 47.8 |
| **Concomitant drugs by ATC^≠^ 4th level therapeutic classes** | | |
| C10AA - HMG CoA reductase inhibitors | 7 | 6.4 |
| B01AC - Platelet aggregation inhibitors excl. heparin | 5 | 4.6 |
| C07AB - Beta blocking agents, selective | 5 | 4.6 |
| C08CA - Dihydropyridine derivatives | 4 | 3.7 |
| N06DA - Anticholinesterases | 4 | 3.7 |
| N06DX - Other anti-dementia drugs | 4 | 3.7 |

* Time from the start of therapy to the date of death; **^≠^** ATC: Anatomical Therapeutic Chemical Classification System

**Table S4.** Main characteristics of cases of deaths for all causes in patients treated with lecanemab submitted to the US FDA Adverse Event Reporting System (FAERS).

| **Report** | **Age (years)/Sex** | **Lecanemab indication** | **LoT**** | **Adverse event(s)** | **TTO (days)*** | **Dechallenge***/Rechallenge***** | **Other drugs** |
| --- | --- | --- | --- | --- | --- | --- | --- |
| 1 | NS / NS | NS | NS | drowning |  | D / NS |  |
| 2 | NS / NS | NS | NS | myocardial infarction |  | D / NS |  |
| 3 | NS / female | NS | 10 months | lung cancer metastatic, death |  | D / NS |  |
| 4 | NS / female | Product used for unknown indication | NS | death |  | D / NS |  |
| 5 | NS / female | NS | NS | cerebral hemorrhage, pulmonary embolism |  | D / NS |  |
| 6 | NS / male | NS | NS | death |  | D / NS |  |
| 7 | 62 / female | NS | NS | fall, head injury, amyloid related imaging abnormality-microhemorrhages and haemosiderin deposits, agitation, dementia alzheimer's type | 148 | D / NS |  |
| 8 | 67 / male | Dementia Alzheimer's type | NS | chills, dyspnoea, ileus, brain natriuretic peptide increased, heart rate decreased, back pain, gastric dilatation, hypokalaemia, back pain, peripheral swelling, nasopharyngitis, hypoxia, electrocardiogram qt prolonged, pulmonary embolism, abdominal rigidity | 265 | N / D |  |
| 9 | 69 / female | Dementia Alzheimer's type | 4 days | cerebral hemorrhage, amyloid related imaging abnormality-oedema/effusion | 54 | D / NS | aspirin |
| 10 | 69 / female | NS | NS | oedema, cerebral mass effect, therapy interrupted, amyloid related imaging abnormality-oedema/effusion, cerebral hemorrhage |  | NS / NS |  |
| 11 | 69 / female | Dementia Alzheimer's type | NS | infusion related reaction, brain oedema, hospice care | 44 | N / D |  |
| 12 | 71 / male | Dementia Alzheimer's type | NS | death, covid-19 | 235 | D / NS |  |
| 13 | 73 / female | NS | NS | hospice care, cerebral hemorrhage, nausea, dizziness |  | NS / NS |  |
| 14 | 74 / male | NS | NS | cerebral hemorrhage | 47 | NS / NS | donepezil, metoprolol |
| 15 | 74 / female | Dementia Alzheimer's type | 1 day | cardiac arrest | 3 | D / NS | amlodipine besylate, losartan potassium, sodium ferrous citrate |
| 16 | 74 / female | NS | NS | sepsis, multiple organ dysfunction syndrome, amyloid related imaging abnormality-microhemorrhages and haemosiderin deposits, amyloid related imaging abnormality-oedema/effusion |  | NS / NS |  |
| 17 | 74 / female | Dementia Alzheimer's type | 15 days | amyloid related imaging abnormality-oedema/effusion, seizure, amyloid related imaging abnormality-microhemorrhages and haemosiderin deposits | 35 | NS / NS |  |
| 18 | 74 / female | Dementia Alzheimer's type | NS | confusional state, dehydration, delirium | 1049 | Y / NS | acetaminophen\butalbital\caffeine, bupropion hydrochloride, buspirone hydrochloride, dimenhydrinate, diphenhydramine hydrochloride, fluticasone propionate, hydrochlorothiazide\lisinopril, loperamide, lorazepam, meclizine hydrochloride, memantine, metoprolol, pravastatin |
| 19 | 74 / male | Dementia Alzheimer's type | NS | diverticulitis, sepsis, aortic aneurysm rupture | 132 | D / NS |  |
| 20 | 75 / female | Cognitive disorder | 71 days | death | 77 | D / NS | amlodipine besylate, atorvastatin calcium, memantine hydrochloride |
| 21 | 75 / female |  | NS | osteoarthritis, bradycardia, myocardial ischaemia, nausea, ventricular fibrillation, electrocardiogram st segment elevation, loss of consciousness, hypotension | 0 | NS / NS |  |
| 22 | 75 / female | Dementia Alzheimer's type | 1 hour | atrioventricular block, bradycardia, cardiac arrest, hypotension, ventricular fibrillation, nausea, electrocardiogram st segment elevation |  | D / D |  |
| 23 | 75 / female | Dementia Alzheimer's type | NS | vasogenic cerebral oedema, amyloid related imaging abnormality-microhemorrhages and haemosiderin deposits, amyloid related imaging abnormality-oedema/effusion, acute kidney injury, aphasia, status epilepticus, metabolic acidosis, sepsis, seizure, loss of consciousness, infusion related reaction, confusional state, organ failure | 57 | D / D |  |
| 24 | 75 / male | Dementia Alzheimer's type | 44 days | glioma | 30 | NS / NS |  |
| 25 | 76 / male | Dementia Alzheimer's type | 850 days | hyponatraemia, interstitial lung disease | 834 | Y / NS |  |
| 26 | 76 / female | Dementia Alzheimer's type | 456 days | myocardial infarction | 461 | D / NS | acetaminophen, acetaminophen\oxycodone hydrochloride, amlodipine besylate, amoxicillin, atorvastatin, biotin, cyclobenzaprine hydrochloride, docusate sodium, estradiol, estrogens, conjugated, gabapentin, hydrochlorothiazide, levothyroxine, metoprolol, naproxen\naproxen sodium, quinapril, vitamins |
| 27 | 76 / female | NS | 43 days | squamous cell carcinoma of lung | 62 | NS / NS |  |
| 28 | 78 / female | Dementia Alzheimer's type | 43 days | hemorrhage | 111 | D / NS |  |
| 29 | 78 / male | NS | 132 days | death | 138 | D / NS |  |
| 30 | 78 / male | NS | NS | asthenia | 262 | D / NS |  |
| 31 | 79 / male | NS | 351 days | hemorrhagic stroke | 350 | NS / NS | aspirin, buspirone hydrochloride, donepezil, memantine, modafinil, tolterodine |
| 32 | 79 / male | Dementia Alzheimer's type | 113 days | drowning | 123 | D / NS |  |
| 33 | 79 / male | Dementia Alzheimer's type | 825 days | pancreatic carcinoma | 861 | NS / NS | amlodipine besylate\irbesartan, celecoxib, donepezil hydrochloride, herbals, ipragliflozin l-proline, pregabalin, sitagliptin phosphate |
| 34 | 80 / male | NS | NS | confusional state, amyloid related imaging abnormality-microhemorrhages and haemosiderin deposits, seizure, urinary incontinence, white blood cell count increased, pyrexia, oedema |  | NS / NS |  |
| 35 | 80 / female | NS | 98 days | myocardial ischaemia | 161 | D / NS | atorvastatin, bazedoxifene, diquafosol tetrasodium, eldecalcitol, rebamipide |
| 36 | 80 / male | Product used for unknown indication | NS | amyloid related imaging abnormality-oedema/effusion, death |  | D / NS |  |
| 37 | 81 / female | NS | NS | hypoxic-ischaemic encephalopathy | 207 | D / NS | budesonide\formoterol\glycopyrrolate, candesartan, doxazosin mesylate, duloxetine, esomeprazole, fursultiamine, glycopyrronium\indacaterol, mirabegron, nicergoline, sucralfate, tocopheryl nicotinate, d-.alpha. |
| 38 | 81 / male | Dementia Alzheimer's type | 15 days | fall | 33 | D / NS | acetaminophen\tramadol hydrochloride, amlodipine besylate, camostat mesylate, candesartan, clostridium butyricum spores strain m-55, fexofenadine hydrochloride, herbals, lemborexant, magnesium oxide, pancrelipase, polaprezinc, pregabalin, tadalafil, |
| 39 | 81 / male | Dementia Alzheimer's type | 15 days | dementia alzheimer's type, fall | 17 | D / NS |  |
| 40 | 82 / male |  | NS | death |  | D / NS |  |
| 41 | 83 / male | Dementia Alzheimer's type | NS | death |  | D / NS |  |
| 42 | 84 / male | NS | 169 days | subdural haematoma, contusion, fall | 182 | NS / NS | cilostazol, diltiazem hydrochloride, folic acid, furosemide, isosorbide dinitrate, methylcobalamin |
| 43 | 84 / female | NS | NS | mental impairment, amyloid related imaging abnormality-microhemorrhages and haemosiderin deposits, wound infection, decreased appetite, confusional state, neurological decompensation, altered visual depth perception, status epilepticus, amyloid related imaging abnormality-oedema/effusion, disorientation, somnolence |  | NS / NS |  |
| 44 | 84 / male | Dementia Alzheimer's type | NS | amyloid related imaging abnormality-microhemorrhages and haemosiderin deposits, death, status epilepticus, amyloid related imaging abnormality-oedema/effusion, superficial siderosis of central nervous system | 42 | D / NS | fenofibrate, fish oil, simvastatin |
| 45 | 86 / female | Dementia Alzheimer's type | 3 years | atrial fibrillation, cardiac failure, acute myocardial infarction | 1063 | N / NS | alprazolam, aspirin, atorvastatin calcium, clopidogrel bisulfate, furosemide, ipratropium, metoprolol succinate, nicotine, nitroglycerin, pantoprazole, potassium chloride, sertraline hydrochloride, trazodone hydrochloride |
| 46 | 87 / female | Dementia Alzheimer's type | 3 years | pancreatic carcinoma, jaundice | 1283 | D / NS | memantine, metoprolol, raloxifene hydrochloride, rivastigmine, sertraline hydrochloride, simvastatin |

**Table S5.** Comparisons between the CLARITY-AD trial and cohort studies from real-world

|  | **Country** | **N** | **Age & Sex**  **(F, %)** | **Diagnosis, n (%)** | ***APOE4 (%)*** | **Contraindications for the treatment** | **Pre-treatment MRI contraindications** | **Adverse events**  ARIA-H (%)  ARIA-E (%)  mH (%)  MH (%) | **Clinical outcomes^§^** | **Drop-out, n (%)** |
| --- | --- | --- | --- | --- | --- | --- | --- | --- | --- | --- |
| **CLARITY-AD [13]** | Multi-national | 1795 | 71.2  907 (50.5) | MCI: 1082 (62.4)  AD: 662 (38.2) | NC: 30.2  ε4/-: 51.5  ε4/ε4: 14.9 | Psychiatric diagnoses/symptoms that could interfere with study procedures  GDS score > 8 at screening  Anticoagulants and ASA if stable for at least 4 weeks | Any MH  Any cortical infarction or stroke  >1 area of SS  >4 MH  >2 lacunar infarction or stroke | ARIA-E: 12.6 (vs 1.7 in PL)  ARIA-H: 17.3 (vs 9.0 in PL) | aMD: −0.45  (−0.67, −0.23) | LECA: 169 (18.8)  PL: 140 (15.6) |
| **Arai 2025 [30]** | Japan | 20 | 73 (9.6)  8 (40) | MCI: 8 (40)  AD 12 (60)  *Based on CDR-GS* | - | - | - | **During LECA**  ARIA-E: 2 (10)  ARIA-H: 2 (10)  **During DONA**  ARIA-E: 1 (5)  ARIA-H: 1 (5) | 3 showed worsening from 0.5 to 1; 17 remained stable | - |
| **Bregman 2025 [38]** | Israel | 86 | 71.99 (8.2)  53 (62) | MCI: 62 (72%)  AD: 24 (28%)  MMSE: 23.96 (2.69) | ε4 carriers:  46 (53) | ε4/ε4 carriers  Medical, neurological, or psychiatric condition  non-AD diagnosis  Any seizure history  Uncontrolled  bleeding disorders  Use of anticoagulants, Immunologic diseases, Recent immunosuppressive therapy | >4 mH (≤10 mm)  Any MH (>10 mm)  SS, VE, >2  lacunar infarcts or territorial stroke  Fazekas 3 subcortical hyperintensities  CAA-ri  Recent stroke or TIA | ARIA-E: 3 (3.5)  ARIA-H mH: 12 (14)  ARIA-H SS: 1 (1.2) | Significant decline in MMSE  compared to baseline  Significant worsening in younger patients | 17 (19.8) |
| **Chen 2025 [31]** | China | 68 | 68.7 (11.2)  50 (73.5) | Based on CDR scores:  MCI: 36.8%  Mild AD: 39.7%  Moderate AD: 22.1% Severe AD: 1.5%  Evidence of episodic memory impairment  CDR≥ 2 was accepted upon informed consent by participant | NC: 38.2  ε4/-: 50  ε4/ε4: 11.8 | Bleeding disorders Anticoagulation therapy (e.g., warfarin) with an international normalized ratio > 1.5 | > 4 cerebral mH (≤10 mm)  Any MH (> 10 mm) Presence of SS | ARIA-E+H: 13.2 (asymptomatic)  Isolated ARIA-H: 4.4% | **CDR-SB** (V1) −0.46  (−0.77, −0.45) (V2) −0.16 (−0.77, −0.04) | 34 (50) |
| **Kang 2025 [32]** | China | 64 | 68.0 (range 61-74)  32 (50) | MCI: 57 (89.1)  AD: 7 (10.9) | NC: 32.8  ε4/-: 62.5  ε4/ε4: 4.7 | - | ≥4 mH (≤10 mm),  MH (>10 mm),  cSS, vasogenic edema, other vascular conditions  severe WM lesions (Fazekas ≥3)  space-occupying lesions (except asymptomatic meningiomas/arachnoid cysts <1 cm) | ARIA-E: 2 (3.1)  ARIA-H: 6 (9.4) | **CDR-SB (6 m)**  2.30 (1.65) vs. 3.16 (1.72)  P = 0.357 | 30 (66.6)* |
| **Li 2025 [33]** | China | 407 | 68.1 (8.7)  275 (67.6) | MCI: 239 (58.7)  Mild AD: 136 (33.4)  Moderate AD: 32 (7.9) | NC: 38.3  ε4/-: 44.0  ε4/ε4: 12.5  n.d.: 5.2 | Moderate dementia stage and on anticoagulant therapies | - | ARIA-E: 16 (5)  ARIA-H: 35 (10.9) | No significant changes in MMSE, CDR, ADL | 328 (80.6)  *79 patients received lecanemab up to 9 months* |
| **Mervosh 2025 [34]** | US | 19 | 72  (range 57–83)  13 (68.4) | - | NC: 15.8  ε4/-: 42.1  ε4/ε4: 42.1 | - | - | ARIA-E: 1 (5)  ARIA-H: 1 (5) | - | - |
| **Paczynski 2025 [36]** | US | 234 (194)** | 74.3 (SD, 6.7)  101 (52) | MCI***: 164 (85)  AD: 30 (15) | NC: 38  ε4/-: 53  ε4/ε4: 8.2 | Exclusion based on AUR *(Cummings 2023)* | Exclusion based on AUR *(Cummings 2023)* | ARIA-E: 29 (15)  ARIA-H: 13 (6.7) | **Estimated rate of change CDR-SB**  1.11 per year | 23 (9.8) |
| **Rosenbloom 2025 [35]** | US | 165 | 72  110 (67) | MCI: 67 (41)  AD: 98 (59) | NC: 30  ε4/-: 58  ε4/ε4: 12 | Use of anticoagulants | Fazekas score >2 | ARIA-E: 13 (8)  ARIA-H: 11 (7) | - | 12 (7.3) |
| **Shields 2024 [37]** | US | 71 | 72 (range 49-90) | MCI: 35 (49.3)  AD: 36 (50.7) | NC: 37  ε4/-: 51  ε4/ε4: 13 | Severe psychiatric or depressive disorders  Poorly controlled immunologic disorders, unstable medical conditions, stroke, TIA,  bleeding disorders, or seizures in the previous 12 months  CAA-ri/amyloid beta-related angiitis  warfarin, vitamin K antagonists, direct oral anticoagulants, heparin, acute thrombolytics,  and clotting disorders | Severe vascular  dementia (severe cerebrovascular changes on MRI or  multiple lacunar strokes or cortical infarcts)  > 4 mH (10 mm or less at the greatest  diameter)  Single MH (greater than 10 mm at the greatest diameter) | ARIA: 12 (24)  ARIA-E: 3 (6)  ARIA-H: 5 (10) | - | - |

aMD: adjusted mean difference; AD: Alzheimer's disease; ASA: [acetylsalicylic acid;](https://www.bing.com/ck/a?!&&p=1cb6c96ec471ff2edc150846d129bc9039a566de31eb201a1c39df64b3954b3aJmltdHM9MTc2MzQyNDAwMA&ptn=3&ver=2&hsh=4&fclid=1b2de397-cb06-6a72-12a5-f743ca006b82&psq=ASA+acid+in+english&u=a1aHR0cHM6Ly9lbi53aWtpcGVkaWEub3JnL3dpa2kvQXNwaXJpbiM6fjp0ZXh0PUFzcGlyaW4lMjAlMjglMkYlMjAlQ0IlODglQzMlQTZzcCUyMCUyOCVDOSU5OSUyOSUyMHIlQzklQUFuJTIwJTJGJTIwJTVCMTAlNUQlMjklMjBpcyxhbmQlMjBpbmZsYW1tYXRpb24lMkMlMjBhbmQlMjBhcyUyMGFuJTIwYW50aXRocm9tYm90aWMuJTIwJTVCMTElNUQlMjA) CAA: cerebral amyloid angiopathy; FU: follow-up; MCI: mild cognitive impairment; MH: macrohemorrhage; mH: microhemorrhage; NC: noncarriers; PET: positron emission tomography; MRI: magnetic resonance imaging; SS: superficial siderosis; cSS: cortical superficial siderosis; MMSE: Mini Mental State Examination score; CDR: Clinical Dementia Rating; CDR-SB: Clinical Dementia Rating-Sum of Boxes; CAA, Cerebral Amyloid Angiopathy; tPA: Tissue-type plasminogen activator; *When approved by the EMA

§ Values in brackets are 95% confidence intervals or standard deviations depending on data reported in the included studies

* 45 participants were evaluated at baseline for cognitive and non-cognitive outcomes. Percentage is calculated on the total of participants evaluated for cognitive and non-cognitive outcomes. Safety population remained unchanged (64).

** Participants receiving at least 4 doses of lecanemab were considered at risk of ARIA. Data in the table refers to those considered at risk of ARIA (n=194)

*** CDR=0.5 was considered as “MCI” or “very mild dementia” while CDR=1 was considered “mild dementia”

**Table S6.** Comparisons between the CLARITY-AD trial and experts Appropriate Use Recommendations (AURs)

| **Criteria** | **CLARITY-AD [13]** | **US AURs [58]** | **France AURs [59]** | **Sud Korean AURs [60]** |
| --- | --- | --- | --- | --- |
| Age | 50-90 | No age-based restrictions | No age-based restrictions | No age-based restrictions  Frailty assessment and standardised geriatric assessment for older participants |
| Diagnosis | MCI or mild AD dementia | MCI or mild AD dementia | MCI or mild AD dementia  (amnestic and non-amnestic presentations) | MCI or mild AD dementia  (amnestic and non-amnestic presentations) |
| Cognitive functions | MMSE 22-30  CDR-GS: 0.5 or 1.0 | MMSE 22-30  Other cognitive screening instrument with a score compatible with early AD | MMSE 22-30 (included sub-threshold values)  CDR-GS: 0.5-1.0  Lawton Instrumental Activities of Daily Living (IADL) scale used in clinical and research contexts to describe a patient's ability to manage complex daily tasks | MMSE 22-30 (included sub-threshold values)  CDR-GS: 0.5-1.0 |
| Positive biomarker for brain amyloid pathology | Positive amyloid PET/CSF | Positive amyloid PET/CSF | Positive blood biomarker using double threshold (ptau217)*  CSF A+/T+  Positive amyloid PET (if inconclusive CSF or blood biomarkers) | Positive amyloid PET/CSF |
| *APOE4* | No restrictions | No restrictions (caution and increased monitoring) | Lecanemab recommended for *APOE4* carriers and heterozygotes | No restrictions (caution and increased monitoring) |
| Comorbidities contraindicated to the treatment | Psychiatric diagnoses/symptoms that could interfere with study procedures  GDS score > 8 at screening | Any neurologic condition that could interfere with study procedures in the participant  Major depression (Less severe depression or depression that is resolving may be permitted) | Multidisciplinary meetings should review comorbid conditions and address the following questions to assess the risk-benefit ratio | Recent history (within 12 months) of TIA or stroke, history of seizures;  Mental illness, major depression;  Presence of immune diseases;  Presence of a bleeding disorder;  Other unstable medical conditions |
| Pre-treatment MRI exclusion criteria | Any MH  Any cortical infarction or stroke  >1 area of SS  >4 MH  >2 lacunar infarction or stroke | Any MH  Any cortical infarction or stroke  >1 area of SS  >4 MH  >2 lacunar infarction or stroke | ≥ 2 lobar mH (Boston v1.5 criteria for probable CAA)  ≥ 5 MH (anywhere in the brain)  ≥ 1 area of cSS (focal or disseminated)  ≥ 1 MH >10 mm  Recent evidence of vasogenic edema  Multiple lacunar infarcts  Severe subcortical white matter hyperintensities (Fazekas 3)  Evidence of ABRA or CAA-ri  Other major intracranial pathology that may cause cognitive impairment | ≥ 2 lobar mH (Boston v1.5 criteria for probable CAA)  ≥ 5 MH (anywhere in the brain)  ≥ 1 area of cSS (focal or disseminated)  ≥ 1 MH >10 mm  Recent evidence of vasogenic edema  Multiple lacunar infarcts  Severe subcortical white matter hyperintensities (Fazekas 3)  Evidence of ABRA or CAA-ri  Other major intracranial pathology that may cause cognitive impairment |
| Contraidicated therapies | Anticoagulants and ASA if stable for at least 4 weeks | Anticoagulants  tPA | Anticoagulants  No absolute contraindication for tPA but need to perform rapid brain MRI to exclude ARIA before use of tPA | Patients on anticoagulants (e.g., warfarin, heparin, or DOACs) require careful assessment. In individuals receiving lecanemab, the decision to administer tPA, heparin, or other anticoagulants should balance potential benefits against the increased risk of harm. |

AD: Alzheimer's disease; ASA: [acetylsalicylic acid;](https://www.bing.com/ck/a?!&&p=1cb6c96ec471ff2edc150846d129bc9039a566de31eb201a1c39df64b3954b3aJmltdHM9MTc2MzQyNDAwMA&ptn=3&ver=2&hsh=4&fclid=1b2de397-cb06-6a72-12a5-f743ca006b82&psq=ASA+acid+in+english&u=a1aHR0cHM6Ly9lbi53aWtpcGVkaWEub3JnL3dpa2kvQXNwaXJpbiM6fjp0ZXh0PUFzcGlyaW4lMjAlMjglMkYlMjAlQ0IlODglQzMlQTZzcCUyMCUyOCVDOSU5OSUyOSUyMHIlQzklQUFuJTIwJTJGJTIwJTVCMTAlNUQlMjklMjBpcyxhbmQlMjBpbmZsYW1tYXRpb24lMkMlMjBhbmQlMjBhcyUyMGFuJTIwYW50aXRocm9tYm90aWMuJTIwJTVCMTElNUQlMjA) CAA: cerebral amyloid angiopathy; MCI: mild cognitive impairment; MH: macrohemorrhage; mH: microhemorrhage; PET: positron emission tomography; MRI: magnetic resonance imaging; SS: superficial siderosis; cSS: cortical superficial siderosis; MMSE: Mini Mental State Examination score; CDR: Clinical Dementia Rating; CDR-SB: Clinical Dementia Rating-Sum of Boxes; CAA, Cerebral Amyloid Angiopathy; tPA: Tissue-type plasminogen activator; *When approved by the EM

**Table S7.** Checklist for prevalence/incidence studies (cohort studies)

|  | **Items** | | | | | | | | |
| --- | --- | --- | --- | --- | --- | --- | --- | --- | --- |
|  | **1** | **2** | **3** | **4** | **5** | **6** | **7** | **8** | **9** |
| **Arai 2025** | N | Y | N | N | Y | Y | Y | N | N |
| **Chen 2025** | Y | Y | Y | N | Y | N | U | U | U |
| **Kang 2025** | Y | Y | Y | Y | Y | Y | Y | Y | Y |
| **Li 2025** | Y | Y | Y | Y | Y | Y | Y | Y | Y |
| **Mervosh 2025** | N | Y | N | N | Y | Y | Y | N | N |
| **Rosenbloom 2025** | Y | Y | Y | Y | N | N | N | N | Y |
| **Paczynski 2025** | Y | Y | Y | Y | Y | Y | Y | Y | Y |
| **Shields 2024** | Y | Y | Y | Y | Y | Y | Y | N | Y |
| **Bregman 2025** | Y | Y | Y | Y | Y | Y | Y | Y | Y |

Y=Yes; N=No; U=Unclear

**Items**

1. Was the sample frame appropriate to address the target population?
2. Were study participants sampled in an appropriate way?
3. Was the sample size adequate?
4. Were the study subjects and the setting described in detail?
5. Was the data analysis conducted with sufficient coverage of the identified sample?
6. Were valid methods used for the identification of the condition?
7. Was the condition measured in a standard, reliable way for all participants?
8. Was there appropriate statistical analysis?
9. Was the response rate adequate, and if not, was the low response rate managed appropriately?

**Table S8.** Checklist for case reports (JBI checklist)

|  | **Items** | | | | | | | |
| --- | --- | --- | --- | --- | --- | --- | --- | --- |
|  | **1** | **2** | **3** | **4** | **5** | **6** | **7** | **8** |
| **Alammar 2025** | Y | Y | Y | Y | Y | Y | Y | Y |
| **Bitar 2025** | Y | Y | Y | Y | Y | Y | Y | Y |
| **Chen 2025** | Y | U | Y | Y | Y | Y | Y | Y |
| **Hanyu 2025** | Y | N | Y | Y | Y | Y | N | Y |
| **Naeshiro 2025** | Y | Y | Y | Y | Y | Y | Y | Y |
| **Navarra 2025** | Y | Y | Y | Y | Y | Y | Y | Y |
| **Schwartz 2025** | Y | Y | Y | Y | Y | Y | Y | Y |
| **Wang Y. 2025*** | Y | Y | Y | Y | Y | Y | Y | Y |
| **Wang X. 2025*** | Y | N | Y | U | Y | Y | NA | N |
| **Zou 2025** | Y | Y | Y | Y | Y | Y | Y | Y |
| **Watanabe 2025** | Y | Y | Y | Y | Y | Y | Y | Y |
| **Gibson 2024** | Y | Y | Y | Y | Y | Y | Y | Y |
| **Ji 2024** | Y | N | Y | U | Y | Y | Y | Y |
| **Shinohara 2024** | Y | N | Y | Y | Y | U | Y | Y |
| **Yamazaki 2024** | Y | Y | Y | Y | Y | Y | Y | Y |
| **Reish 2023** | N | N | N | N | Y | Y | Y | Y |
| **Solopova 2023** | Y | Y | Y | Y | Y | Y | Y | Y |

Y=Yes; N=No; U=Unclear; NA=Not applicable; * Two cases investigated

**Items**

1. Were patient’s demographic characteristics clearly described?
2. Was the patient’s history clearly described and presented as a timeline?
3. Was the current clinical condition of the patient on presentation clearly described?
4. Were diagnostic tests or assessment methods and the results clearly described?
5. Was the intervention(s) or treatment procedure(s) clearly described?
6. Was the post-intervention clinical condition clearly described?
7. Were adverse events (harms) or unanticipated events identified and described?
8. Does the case report provide takeaway lessons?
