# Supplementary material for: Regulatory, clinical, and post-marketing challenges of lecanemab for Alzheimer’s disease: insights from real-world data
Source: Neurol Sci. 2026 Mar 6;47(4):322. doi: 10.1007/s10072-026-08829-4 (PMC12963267; doi:10.1007/s10072-026-08829-4)
Supplement: Supplementary file 2 — Supplementary file2 (PDF 209 KB) [file 10072_2026_8829_MOESM2_ESM.pdf]

Figure S1. PRISMA flow diagram of included studies (literature updated to November 16, 2025)

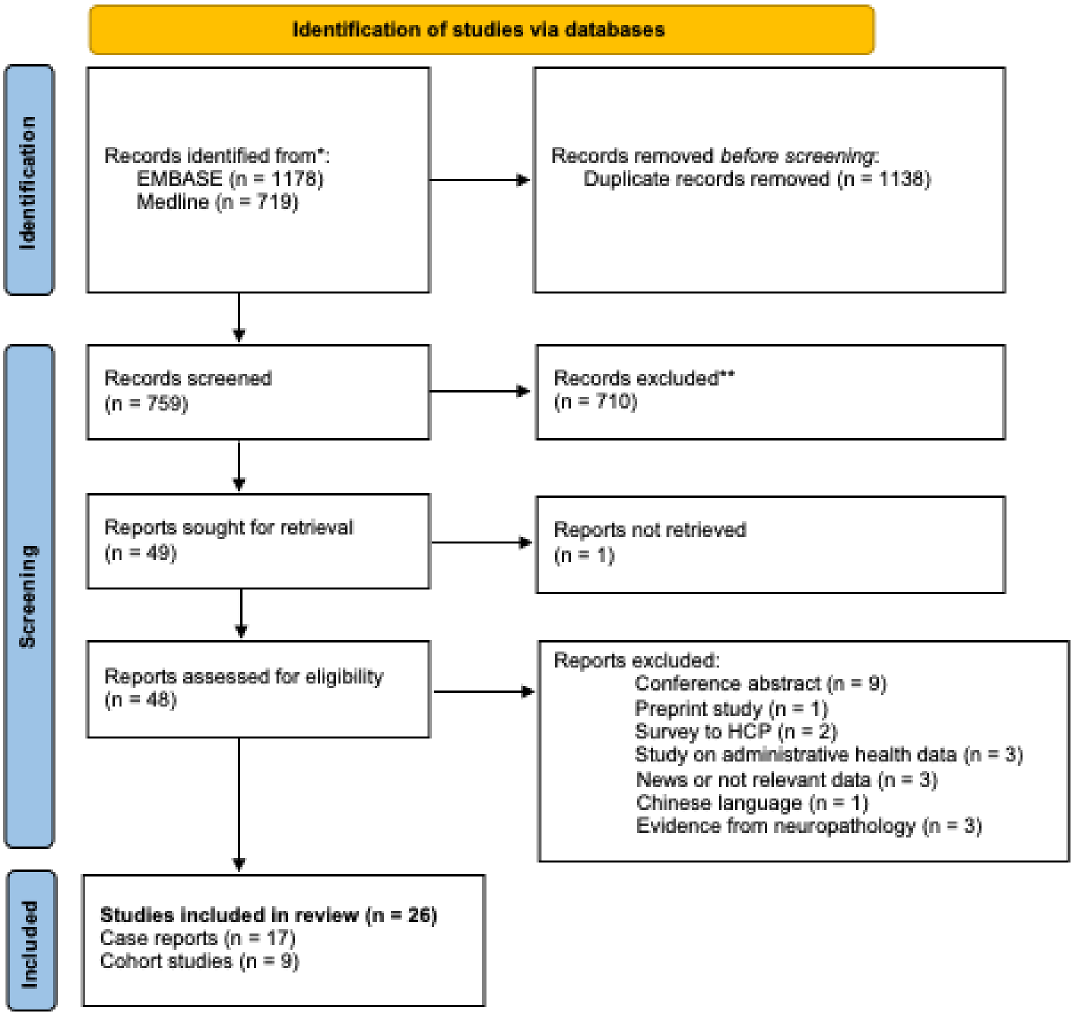

*Consider, if feasible to do so, reporting the number of records identified from each database or register searched (rather than the total number across all databases/registers).

**If automation tools were used, indicate how many records were excluded by a human and how many were excluded by automation tools.

Source: Page MJ, et al. BMJ 2021;372:n71. doi: 10.1136/bmj.n71.

This work is licensed under CC BY 4.0. To view a copy of this license, visit https://creativecommons.org/licenses/by/4.0/
